# K^+^/Pb^2+^ ion exchange-induced structural transitions of G-quadruplexes under molecular crowding conditions

**DOI:** 10.1039/d6ra03491h

**Published:** 2026-07-06

**Authors:** Sumin Kim, Hee Chang Kwon, Yoon Jung Jang, Young-Hun Kim, Ji Hoon Han

**Affiliations:** a Department of Chemical and Biological Engineering, Gyeongkuk National University 1375 Gyeongdong-ro Andong Gyeongbuk 36729 Republic of Korea jhan@gknu.ac.kr; b College of Basic Education, Yeungnam University Gyeongsan Gyeongbuk 38541 Republic of Korea; c Department of Environmental Engineering, Gyeongkuk National University 1375 Gyeongdong-ro Andong Gyeongbuk 36729 Republic of Korea

## Abstract

Because biochemical experiments are typically performed under dilute conditions despite the highly crowded nature of the cellular environment, crowding agents such as PEG are used to mimic intracellular conditions and investigate biomolecular stability and structural transitions. In this study, we systematically investigated the effect of molecular crowding on the ion exchange between K^+^ and Pb^2+^ in four representative G-quadruplexes, TBA, HTG, PS2.M, and T2, using PEG200 as a crowding agent. Circular dichroism (CD), fluorescence (FL), and thermal melting analyses revealed that the extent of Pb^2+^ induced conformational change and stability strongly depended on both sequence and crowding conditions. In contrast to dilute conditions, the order of structural variation by Pb^2+^ under molecular crowding shifted to TBA > HTG > PS2.M > T2. TBA exhibited the greatest conformational flexibility and Pb^2+^ affinity, whereas PS2.M showed a comparatively increased binding affinity relative to the other sequences as the degree of molecular crowding increased. These results highlight that molecular crowding modulates ion-induced structural transitions of G-quadruplexes, providing new insights into G-quadruplex behavior under physiologically relevant conditions.

## Abstract 2

Molecular crowding reshapes Pb^2+^-mediated G-quadruplex structural transitions, thermal stability, and apparent binding behavior in a sequence-dependent manner.

## Graphical abstract

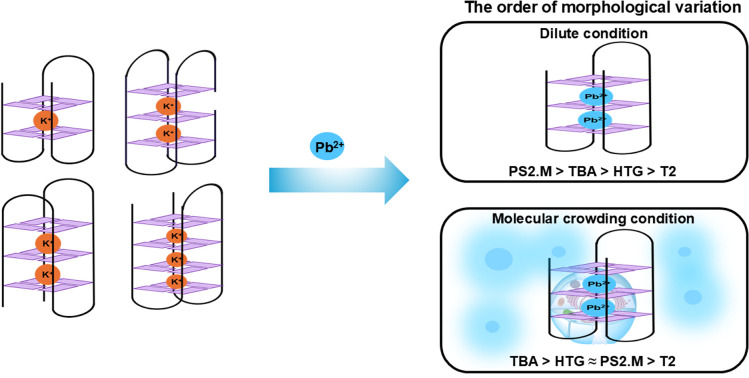

## 1. Introduction

Most biochemical research performed in dilute conditions is predicted to represent those in living cells. However, living cells contain various biomolecules including proteins, nucleic acids, lipids, and polysaccharides. The *in vivo* concentration of biomolecules in a cell is between 50 and 400 g L^−1^.^1–3^ To investigate feasible biochemical research under cellular conditions, many experiments have been performed using crowding agents such as polyethylene glycol (PEG), dextran, sucrose, ficoll, and ethylene glycol. Many studies demonstrated that molecular crowding conditions affect biomolecular behavior such as conformation, stability, and functions compared with dilute conditions.^4,5^ For instance, under molecular crowding conditions, the stability of DNA duplexes as canonical structures decreases, whereas that of G-quadruplexes as non-canonical structures increases.^6^ Particularly, G-quadruplexes exhibit structural polymorphism dependent on various cellular environments.^7,8^

G-quadruplex, in which four guanine bases are connected *via* Hoogsteen base pairing in the same plane to form a G-quartet in the presence of certain mono-cation, can be formed by associations of guanine-rich sequences.^9–11^ Over 370 000 guanine-rich sequences were reported by bioinformatic researchers. Importantly, these guanine-rich sequences are located at important genome regions including oncogene regulatory regions, ribosomal DNAs, and the telomeric ends of chromosomes.^12,13^ The extensive studies have demonstrated that G-quadruplex can play crucial roles such as transcription, gene expression, telomere maintenance, promoter activation, and gene arrangement.^14,15^

A variety of metal ions can influence the stability and topology of G-quadruplexes.^16,17^ Among them, Pb^2+^, one of the most hazardous environmental pollutants, has been reported to strongly interact with several sequences that form G-quadruplex structures at micromolar concentrations.^18–20^ Previous studies have demonstrated that Pb^2+^ can bind to specific G-quadruplex structures, often more strongly than K^+^, and induce characteristic CD spectral changes, thermal stability shifts, and high affinity binding responses. These interactions have been widely utilized for the development of Pb^2+^ responsive G-quadruplex based sensors and ion responsive DNA systems. In addition, Pb^2+^ exposure is associated with adverse effects on the nervous system, kidneys, blood pressure regulation, and hematological function, with children being particularly susceptible because of their greater sensitivity and higher absorption rates.^21,22^

Although previous studies have established strong interactions between Pb^2+^ and specific G-quadruplexes, most have focused on individual Pb^2+^ binding G-quadruplex sequences or Pb^2+^ sensing applications under dilute conditions.^23–39^ Furthermore, although molecular crowding is known to alter G-quadruplex conformation and stability, the effect of PEG induced molecular crowding on G-quadruplex structural transitions caused by the replacement of K^+^ with Pb^2+^ has not been systematically investigated. It remains unclear how Pb^2+^ exchange and molecular crowding individually and collectively affect G-quadruplex conformation, thermal stability, and apparent Pb^2+^ binding affinity. To the best of our knowledge, this is the first comparative study to examine how molecular crowding modulates Pb^2+^ driven structural transitions, thermal stability, and apparent binding behavior across representative Pb^2+^ sensitive G-quadruplex sequences.

Herein, we systematically investigated how PEG200 induced molecular crowding affects structural transitions caused by the replacement of K^+^ with Pb^2+^ in four representatives Pb^2+^ sensitive G quadruplex sequences, including TBA, HTG, PS2.M, and T2. By combining CD spectroscopy, thermal melting analysis, thioflavin-T (ThT) fluorescence displacement assay, and apparent binding analysis, we evaluated the sequence dependent effects of molecular crowding on G-quadruplex conformation, thermal stability, and Pb^2+^ binding behavior during K^+^ to Pb^2+^ exchange. To the best of our knowledge, this is the first systematic comparative study to examine how molecular crowding modulates Pb^2+^ driven structural transitions, thermal stability, and apparent binding behavior across multiple G quadruplex sequences. This approach reveals that, under molecular crowding conditions, the structural, thermal, and binding responses of G-quadruplexes to Pb^2+^ are strongly dependent on sequence identity.

## 2. Experimental

### 2.1. Materials and reagents

TBA(5′-GGTTGGTGTGGTTGG-3′), HTG(5′-AGGGTTAGGGTTAGGGTTAGGG-3′), PS2.M(5′-GTGGGTAGGGCGGGTTGG-3′), T2(5′-GGGGTTGGGGTTGGGGTTGGGG-3′), oligonucleotides its complimentary sequence were purchased from Bioneer (Korea). Potassium chloride and lead(ii) nitrate were procured from Sigma-Aldrich. Polyethylene glycol 200 (PEG200) were purchased from TCI (Tokyo Chemical Industry, Japan). Tris(hydroxymethyl)aminomethane base was purchased from TCI. thioflavin T (ThT) were purchased from ACROS ORGANICS product of India.

### 2.2. Circular dichroism (CD) spectroscopy measurement

3 µM Oligonucleotides were annealed in 20 mM Tris–HCl buffer containing KCl (5, 10, or 20 mM) at 95 °C for 5 min. After the annealing process, the samples were slowly cooled to room temperature over 4 to 5 hours. Some samples were added with Pb(NO_3_)_2_ and incubated at 37 °C for 1 h. Circular dichroism experiments were performed on J-810 spectropolarimeter at room temperature (25 °C): wavelength: 220 nm to 400 nm; data interval: 1 nm; band width: 2 nm; scan speed: 50 nm min^−1^; response time: 2 s.

Conformational index, *r* value, used to assess the transition toward a Pb^2+^-associated G-quadruplex conformation, and was calculated based on a modified form of the previous equation that is typically characterized by CD spectra. In general, the two peaks at 265 nm and 295 nm were used to distinguish different conformations of G-quadruplex, while the peak at 314 nm was indicative of a Pb^2+^-associated G-quadruplex conformation when G-quadruplex was bound to Pb^2+^. The *r* value was determined by the following equation:
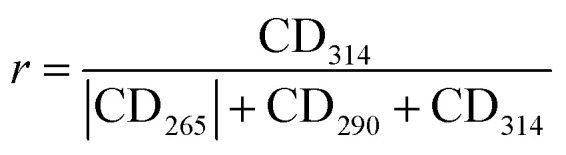
where CD_265_, CD_290_, and CD_314_ are the molar dichroic absorption of peaks at 265, 290, and 314 nm, respectively.

### 2.3. Fluorescence quenching experiment

The solution of 3 µM DNA was annealed with 10 mM KCl, and 6 µM ThT was added to the solution in the presence of 20 mM Tris–HCl buffer (pH 7.0). The sample was incubated for 1 h after the addition of 10 µM Pb(NO_3_)_2_. Fluorescence experiments were performed on SCINCO FS-2 spectrometer at room temperature (25 °C). PMT voltage: 500 V; scan speed: 600 nm min^−1^; integration: 20; response time: 1.0 s; excited slit and emission slit: 5 nm; exited wave: 420 nm; emission wavelength: 450 to 650 nm. Quenching efficiency was calculated by using the following equation:
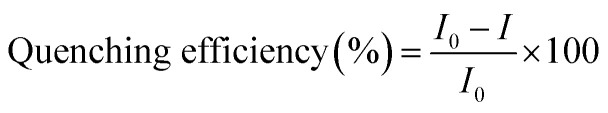
where *I*_0_ and *I* are the emission intensities of ThT bound to G-quadruplex in the absence and the presence of Pb^2+^.

### 2.4. Melting temperature measurement

The samples containing 3 µM oligonucleotide, 20 mM Tris–HCl, and either 5 mM KCl or 10 µM Pb(NO_3_)_2_, together with an appropriate amount of PEG200 (w/w), were annealed at 95 °C for 5 min and then slowly cooled to room temperature over 4–5 h. To replace K^+^ with Pb^2+^ in the G-quadruplex, Pb(NO_3_)_2_ was added to the samples containing G-quadruplexes folded in 5 mM K^+^ after annealing.

The melting temperature curve was obtained using JASCO V-730 spectrometer equipped with a temperature control accessory (JASCO PAC-743) and 10 mm micro auto eight-cell holder. The sample was measured from 20 °C to 95 °C selected wavelength: 295 nm; data interval: 1.0 °C; wait time: 60 s; temperature gradient: 1 °C min^−1^. The *T*_m_ values of the melting curves were calculated by V-730 software.

### 2.5. Determination of binding affinity for Pb^2+^

The samples containing 3 µM DNA, 20 mM Tris–HCl, 10 mM KCl, and an appropriate amount of PEG200 (w/w) were heated to 95 °C for 5 min and then slowly cooled to room temperature over 4–5 h. After cooling down to room temperature, 10 µM Pb(NO_3_)_2_ was added to each sample, mixed gently, and incubated for 5 min at room temperature prior to CD spectral measurements.

To quantitatively evaluate the binding affinity, the binding constant (*K*_a_) of Pb^2+^ toward each G-quadruplex was determined based on the Langmuir adsorption model. The equilibrium binding response was obtained from the change in circular dichroism (CD) signal intensity (Δ*A*, in milli-degrees at 314 nm), which reflects the extent of Pb^2+^ association with the G-quadruplex. The relationship between Pb^2+^ concentration and the equilibrium response was fitted according to the following equation:
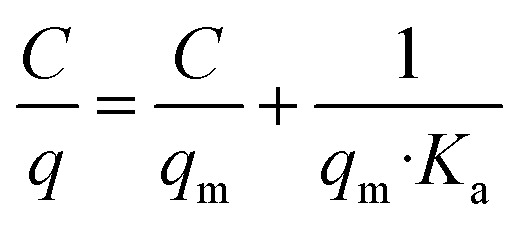
where *q* is the CD signal intensity at 314 nm, and *C* is the Pb^2+^ concentration. *K*_a_ is the apparent binding constant, and *q*_m_ is the CD signal intensity when *C* is infinity. The binding constant was determined using data obtained in duplicate.

## 3. Results and discussion

### 3.1. Effects of sequence variation on CD change of G-quadruplex by Pb^2+^ under different concentrations of K^+^

**Fig. 1:**
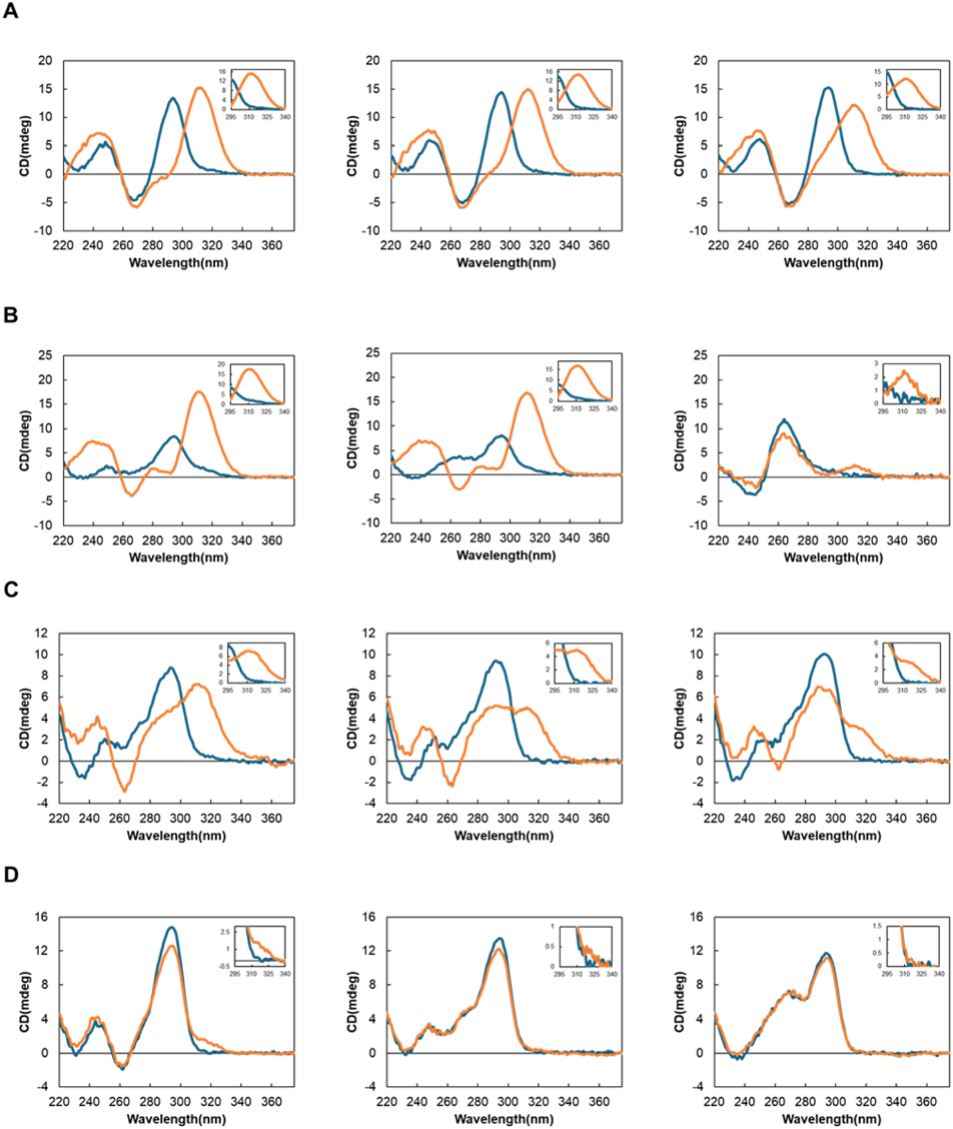
CD spectra of various G-quadruplexes, with TBA (A), PS2.M (B), HTG (C), and T2 (D), containing different concentrations of K^+^ (blue line) and affected by 10 µM Pb^2+^ (orange line) under diluted conditions. 3 µM of DNA was used and the samples contain 5 mM K^+^ (left panel), 10 mM K^+^ (middle panel), and 20 mM K^+^ (right panel). All samples contain 20 mM Tris–HCl buffer (pH 7.0). Insets show magnified views of the CD spectral region around 314 nm to highlight Pb^2+^-associated CD signal changes.

To investigate the structural changes of G-quadruplexes under different concentration of K^+^, we measured the CD spectra of well-known Pb^2+^-responsive sequences, including TBA, HTG, PS2.M, and T2 G-quadruplex (Fig. 1A–D). Considering the strict dependence of G-quadruplex stability on K^+^, K^+^ concentration was carefully controlled within the range of 5 mM to 20 mM. The CD spectrum of TBA at 5, 10, and 20 mM exhibited a negative band at 265 nm and a positive band at 295 nm, indicating the anti-parallel conformation of the G-quadruplex.^40,41^ On the other hand, the CD spectra of HTG and T2 exhibited a positive band at 295 nm with a shoulder at 270 nm, suggesting a hybrid G-quadruplex conformation.^42^ As the K^+^ concentration increased to 10 and 20 mM, both sequences showed an increase in positive ellipticity at 270 nm. For PS2.M G-quadruplex, a similar increase in ellipticity at 270 nm was observed, while a distinct positive band at 265 nm appeared under 20 mM K^+^, indicating that a conformation was changed to parallel structure. In addition, *r*′ value as the conformation index exhibited a consistent trend across the results (Fig. S2).^43^

**Fig. 2:**
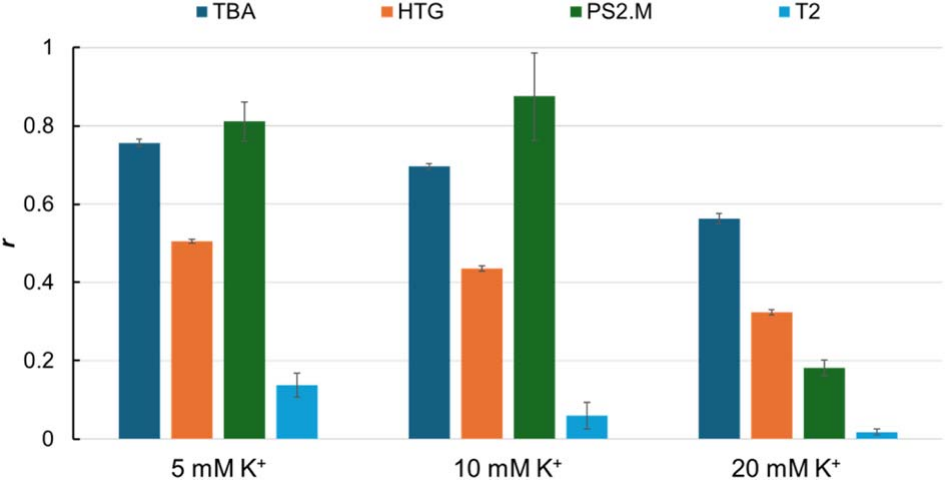
Values of the conformation index, *r* value, for various G-quadruplexes, with TBA (dark blue), HTG (orange), PS2.M (green), and T2 (blue), used to assess the transition toward a Pb^2+^-associated G-quadruplex conformation. The *r* value was calculated from the CD results above using the equation described in the Materials and methods section. The *r* values were calculated from measurements performed in duplicate.

Upon the addition of Pb^2+^, the CD spectrum of each G-quadruplex exhibited a positive band at around 314 nm, suggesting Pb^2+^-associated G-quadruplex structural modulation (Fig. 2).^23–38^ A positive CD band around 314 nm was consistently observed in our Pb^2+^-bound G-quadruplexes, although CD spectroscopy alone cannot unambiguously determine the precise topology of G-quadruplexes. In the absence of K^+^, Pb^2+^-bound TBA, HTG, PS2.M, and T2 all exhibited a positive CD band around 314 nm, consistent with previous studies reporting characteristic positive CD signals near 312–314 nm for Pb^2+^-bound G-quadruplexes (Fig. S1). Therefore, the increase in the CD signal around 314 nm was used as an empirical spectral indicator to compare Pb^2+^-associated G-quadruplex structural modulation among different sequences and crowding conditions. To distinguish and quantify this conformational change, we used a modified index, denoted as the *r* value. As the K^+^ concentration increased from 5 mM to 20 mM, the *r* values gradually decreased, suggesting that the transition of TBA, HTG, and T2 G-quadruplex topology to the Pb^2+^-associated G-quadruplex conformation gradually decreased. In contrast, the *r* value for PS2.M showed a significant decrease. This might be attributed to stable conformation of parallel structures that were formed at high K^+^ concentration. The *r* value for PS2.M under 10 mM showed over 0.80 while that for TBA showed 0.70 under the concentration of 10 mM K^+^. The order of morphological variation induced by Pb^2+^ is PS2.M > TBA > HTG > T2. These results indicate that PS2.M G-quadruplex exhibits the greatest degree of structural rearrangement at 10 mM K^+^, whereas TBA G-quadruplex exhibits the highest degree at 20 mM K^+^.

### 3.2. Effects of sequence variation on CD change of G-quadruplex by Pb^2+^ under various molecular crowding conditions

**Fig. 3:**
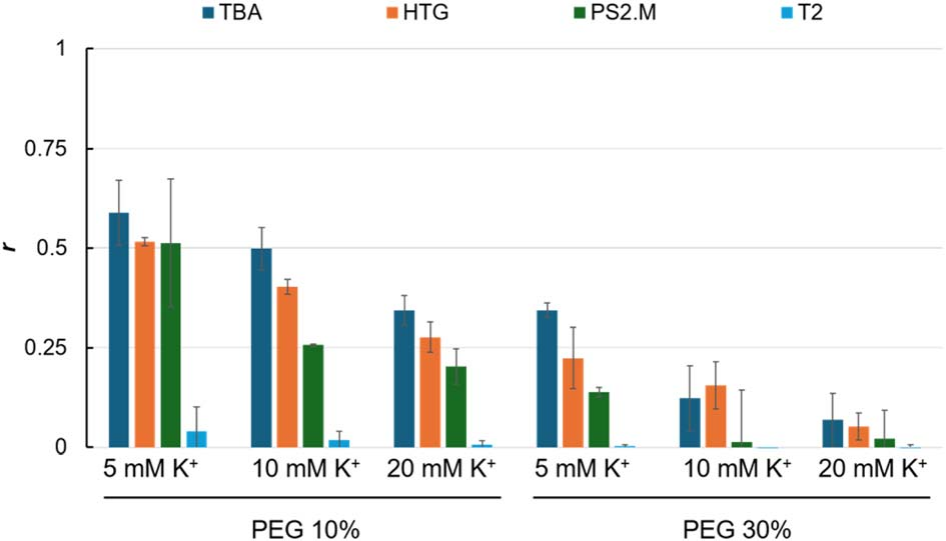
Values of the conformation index, *r* value, for various G-quadruplexes, with TBA, HTG, PS2.M, and T2, used to assess the transition toward a Pb^2+^-associated G-quadruplex conformation under 10 wt% and 30 wt% PEG200. 3 µM DNA was used with appropriate concentration of K^+^. All samples contain appropriate concentration of 20 mM Tris–HCl buffer (pH 7.0). The *r* values were calculated from measurements performed in duplicate.

In order to explore the structural change of G-quadruplex under molecular crowding conditions, we measure the CD spectra for each G-quadruplex containing different cations in the presence of 10 wt% and 30 wt% PEG200 and calculate the *r* value (Fig. 3 and S3–S6). All *r* values for each G-quadruplex under molecular crowding conditions decreased in comparison to those under diluted conditions. Although G-quadruplex structural changes under molecular crowding conditions were less pronounced than under diluted conditions, PS2.M, TBA and HTG G-quadruplexes in the presence of 5 mM K^+^ and 10 wt% PEG200 showed a topology change to a Pb^2+^-associated G-quadruplex conformation in over 50% of the population. TBA G-quadruplex in the presence of 5 mM K^+^ and 10 wt% PEG200 exhibited a structural change in approximately 59% of the population. Moreover, regardless of different concentration of PEG200, the degree of topological change in TBA G-quadruplex under molecular crowding conditions was greater than that in PS2.M, suggesting that TBA G-quadruplex possesses more structural flexibility than other G-quadruplexes, allowing more pronounced conformational rearrangements under molecular crowding conditions. Similarly, HTG G-quadruplex exhibited more pronounced structural changes than PS2.M under 10 wt% PEG200 across the range of 5–20 mM K^+^, and furthermore under 30 wt% PEG200 in the presence of both 5 mM and 10 mM K^+^. In addition, fluorescence quenching experiments were conducted based on the displacement of thioflavin T (ThT) upon Pb^2+^-induced G-quadruplex topology changes (Fig. S7–S11). Although HTG G-quadruplex exhibited higher fluorescence quenching at 5 mM K^+^ with 30 wt% PEG200 than at 20 mM K^+^ with 10 wt% PEG200, due to variations in ThT binding affinity depending on G-quadruplex conformation, the overall trend was similar to that observed in the CD analysis. The TBA G-quadruplex exhibited the greatest fluorescence quenching under both 10 and 30 wt% PEG200, suggesting a pronounced conformational change, whereas T2 showed minimal variation.

Based on CD and FL results, the order of G-quadruplex topological change is TBA > HTG ≈ PS2.M > T2. TBA G-quadruplex has two G quartet layers, whereas HTG and PS2.M have three, and T2 has four, rendering TBA G-quadruplex more susceptible to Pb^2+^ induced topological changes under molecular crowding conditions.^44–46^

### 3.3. Effects of thermal stability for G-quadruplex with various cations under molecular crowding conditions

**Fig. 4:**
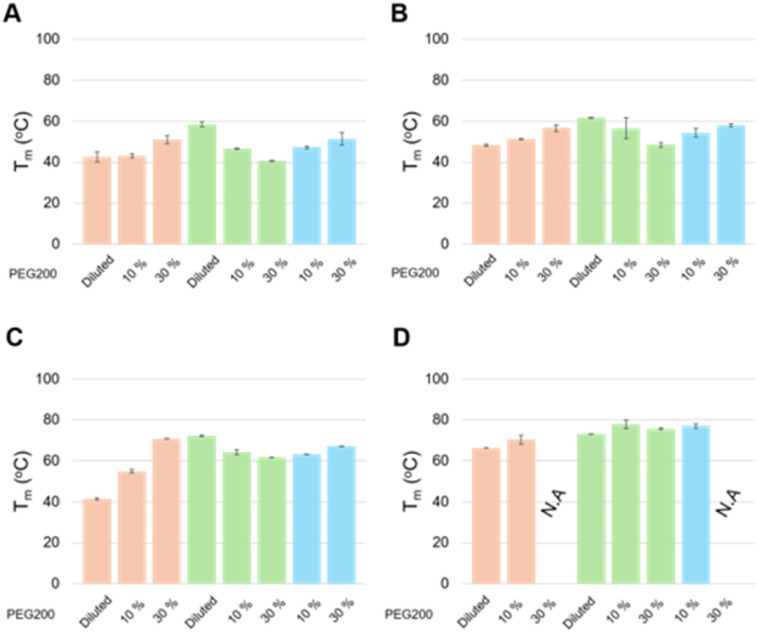
Histograms of the melting temperatures of G-quadruplexes for TBA (A), HTG (B), PS2.M (C), and T2 (D) under diluted and molecular crowding conditions (PEG200, 10% and 30%). Colored bars represent different ionic conditions: 5 mM K^+^ only (red), 10 µM Pb^2+^ only (green), and 5 mM K^+^ with 10 µM Pb^2+^ (blue). All samples contained 3 µM DNA with appropriate concentrations of cations and PEG200 in the presence of 20 mM Tris–HCl buffer (pH 7.0). All experiments were performed in duplicate.

To evaluate the effect of Pb^2+^ on G-quadruplex stability under molecular crowding conditions, we measured the melting temperature (*T*_m_) of each G-quadruplex (Fig. 4 and S12–S14). All K^+^-containing G-quadruplexes exhibited increased *T*_m_ values as the PEG200 concentration increased, indicating that PEG200-induced molecular crowding enhanced the thermal stability of K^+^-stabilized G-quadruplexes. However, the *T*_m_ values of Pb^2+^-containing TBA, HTG, and PS2.M decreased with increasing PEG200 concentration, whereas that of Pb^2+^-containing T2 slightly increased under molecular crowding conditions (Table S1).

The different responses of G-quadruplexes to K^+^ and Pb^2+^ can be partly explained by their distinct ionic properties in the G-quartet cavity.^30^ K^+^ is generally considered an efficient stabilizing monovalent cation because it fits well into the central G-quartet channel and coordinates with guanine O6 atoms. In contrast, Pb^2+^ is a divalent cation with a higher charge density and a different coordination preference. Previous structural studies have shown that Pb^2+^ can coordinate more tightly with guanine O6 atoms than K^+^, resulting in shorter metal-O6 distances and more compact G-quartet arrangements. These differences may allow Pb^2+^ to stabilize certain G-quadruplex sequences more strongly than K^+^. However, Pb^2+^ may also induce sequence-dependent conformational rearrangement or destabilization depending on the crowding environment.

According to a previous study by Li's group, parallel G-quadruplexes were stabilized under crowding conditions in the presence of Pb^2+^, whereas PEG exhibited minimal or even destabilizing effects on antiparallel and hybrid G-quadruplexes.^38^ Similar to these previous results, the stability of the parallel T2 G-quadruplex was enhanced by PEG200, whereas those of the antiparallel TBA and hybrid HTG G-quadruplexes were reduced. Interestingly, the parallel PS2.M G-quadruplex exhibited reduced stability under molecular crowding conditions, in contrast to the previous result. This may be attributed to increased dehydration or local strain around the unlinked stem region, because PS2.M contains one stem that is not fully linked.

To further understand the thermodynamic basis of K^+^ to Pb^2+^ exchange under molecular crowding conditions, van't Hoff analysis was performed to extract Δ*H*° and Δ*S*° values from the melting curves (Table S2). Under K^+^ only and Pb^2+^ only conditions, the Δ*H*° and Δ*S*° responses were strongly sequence dependent, indicating that PEG200 affects G-quadruplex unfolding differently depending on both the cation and sequence.

Importantly, the K^+^ to Pb^2+^ exchange condition showed thermodynamic behavior distinct from direct Pb^2+^ folding. For TBA and HTG, the exchange condition showed higher *T*_m_ values than the Pb^2+^ only condition under PEG200 crowding, suggesting that Pb^2+^ addition to K^+^ formed G-quadruplexes generated more stable exchange states. For PS2.M, the exchange state showed intermediate thermodynamic behavior between the K^+^ only and Pb^2+^ only states. In particular, the decreased Δ*H*° and Δ*S*° at 30 wt% PEG200 suggests that Pb^2+^ addition reorganized the K^+^ formed PS2.M structure rather than simply preserving the strongly stabilized K^+^ bound state. For T2, it is difficult to interpret thermodynamic results due to the exchange conditions maintained very high thermal stability.

Overall, these results indicate that Pb^2+^ addition to K^+^ formed G-quadruplexes under PEG200 induced molecular crowding generates sequence dependent exchanged states with thermodynamic properties distinct from direct Pb^2+^ folding. The changes in *T*_m_, Δ*H*°, and Δ*S*° suggest that K^+^ to Pb^2+^ exchange modulates cation coordination, hydration, and conformational entropy around the G-quartet in a sequence dependent manner.

### 3.4. Determination of binding association constants of Pb^2+^ to G-quadruplexes

**Fig. 5:**
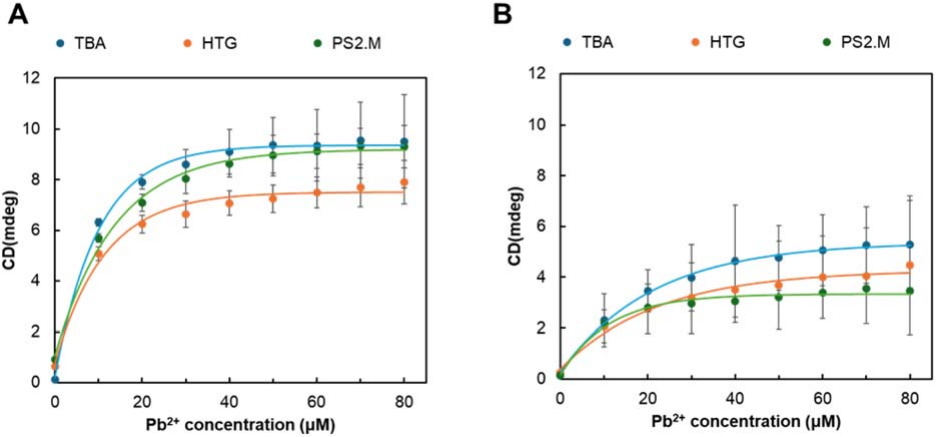
Pb^2+^ concentration-dependent CD spectra of various G-quadruplexes for TBA (blue dot), HTG (orange dot), and PS2.M (green dot) under 10 wt% (A) and 30 wt% PEG200 (B). 3 µM DNA containing 10 mM K^+^ was used and titrated with various concentrations of Pb^2+^. All samples contain appropriate concentration of 20 mM Tris–HCl buffer (pH 7.0). The plotted data represents the average of three independent measurements.

To gain deeper insight into Pb^2+^ binding to various G-quadruplexes under molecular crowding conditions, CD changes were monitored by titrating Pb^2+^ at increasing concentrations (Fig. 5A and B). Under 10 wt% and 30 wt% PEG200 conditions, TBA, HTG, and PS2.M showed gradual increases in CD intensity upon Pb^2+^ addition, whereas T2 was negligibly affected by Pb^2+^ (Fig. S15 and S16). Under 10 wt% PEG200, TBA exhibited the most pronounced CD enhancement in the range of 10–20 µM Pb^2+^, suggesting a strong Pb^2+^-associated CD response compared with the other G-quadruplexes. PS2.M showed a similarly strong but slightly slower increase, whereas HTG exhibited the weakest response. This trend is consistent with previous reports from Kang's groups, in which the extent of Pb^2+^-induced variation in G-quadruplex structure under dilute conditions followed the order TBA > PS2.M > HTG.^39^

Based on the apparent binding constants, *K*_a_, calculated using the Langmuir adsorption model, the *K*_a_ values for TBA, HTG, and PS2.M under 10 wt% PEG200 were 2.86 × 10^5^ M^−1^, 2.15 × 10^5^ M^−1^, and 1.99 × 10^5^ M^−1^, respectively (Fig. S17). Under 30 wt% PEG200, the *K*_a_ values for TBA, HTG, and PS2.M were 9.87 × 10^4^ M^−1^, 9.84 × 10^4^ M^−1^, and 2.02 × 10^5^ M^−1^, respectively. Therefore, the apparent Pb^2+^ binding affinities of TBA and HTG decreased under stronger molecular crowding conditions, whereas PS2.M retained a relatively high *K*_a_ value.

This result indicates that the effect of molecular crowding on apparent Pb^2+^ binding is strongly sequence dependent. Although PS2.M showed a smaller CD intensity change than TBA, its apparent Pb^2+^ binding affinity was maintained under 30 wt% PEG200. This may be related to the compact parallel-like structure of PS2.M, which can support Pb^2+^ coordination with relatively limited global structural perturbation. This interpretation is consistent with the thermal melting and van't Hoff analyses, where the K^+^ to Pb^2+^ exchange state of PS2.M maintained relatively high *T*_m_ but showed decreased Δ*H*° and Δ*S*° under 30 wt% PEG200. Therefore, the relatively high apparent binding affinity of PS2.M under strong crowding may reflect a crowding-induced reorganized exchange state rather than a simple increase in Pb^2+^-induced CD intensity.

## 4. Conclusions

This study elucidated how PEG200-induced molecular crowding affects K^+^ to Pb^2+^ exchange in representative G-quadruplex sequences, including TBA, HTG, PS2.M, and T2. The results show that Pb^2+^-associated G-quadruplex structural modulation, thermal stability, and apparent Pb^2+^ binding behavior are strongly dependent on both sequence identity and molecular crowding conditions.

Under dilute conditions containing K^+^, the extent of Pb^2+^-induced structural variation followed the order PS2.M > TBA > HTG > T2, whereas under molecular crowding conditions this order shifted to TBA > HTG ≈ PS2.M > T2. TBA exhibited the greatest conformational flexibility under crowding conditions, whereas T2 showed minimal structural variation. Thermal melting and van't Hoff analyses further showed that K^+^ to Pb^2+^ exchange produced sequence-dependent exchanged states with thermodynamic properties distinct from direct Pb^2+^ folding. These changes in *T*_m_, Δ*H*°, and Δ*S*° suggest that molecular crowding modulates cation coordination, hydration, and conformational entropy around the G-quartet.

The apparent binding analysis showed that Pb^2+^ binding behavior also varied among G-quadruplex sequences under molecular crowding conditions. In particular, PS2.M retained a relatively high apparent Pb^2+^ binding affinity under 30 wt% PEG200 compared with TBA and HTG. Therefore, this work provides an *in vitro* basis for understanding how molecular crowding modulates metal ion-mediated G-quadruplex structural changes, thermal stability, and apparent binding behavior. These findings establish a useful model framework for studying metal ion-mediated G-quadruplex modulation under crowded environments and provide a basis for future investigations in more complex biological systems.

## Conflicts of interest

There are no conflicts to declare.

## Supplementary Material

RA-OLF-D6RA03491H-s001

## Data Availability

The data supporting this article have been included as part of the supplementary information (SI). Additional raw data are available from the corresponding author upon reasonable request. Supplementary information: additional experimental details, supporting data, and supplementary figures and tables. See DOI: https://doi.org/10.1039/d6ra03491h.
