# Supplementary material for: K+/Pb2+ ion exchange-induced structural transitions of G-quadruplexes under molecular crowding conditions
Source: RSC Adv. 2026 Jul 6;16(36):37816–22. doi: 10.1039/d6ra03491h (PMC13334766; doi:10.1039/d6ra03491h)
Supplement: Supplementary file 1 [file RA-016-D6RA03491H-s001.pdf]

Supplementary Information (SI) for RSC Advances.
This journal is © The Royal Society of Chemistry 2026

Supporting Information

K^+/Pb^{2+} Ion Exchange-Induced Structural Transitions of G-Quadruplexes under Molecular Crowding Conditions

Sumin Kim,[a] Hee chang Kwon,[a] Yoon Jung Jang,[b] Young-Hun Kim[c] and Ji Hoon Han[a,*]

[a] Department of Chemical and Biological Engineering, Andong National University, 1375 Gyeongdong-ro, Andong, Gyeongbuk, Republic of Korea, 36729.

[b] College of Basic Education, Yeungnam University, Gyeongsan, Gyeongbuk, Republic of Korea, 38541.

[c] Department of Environmental Engineering, Gyeongkuk National University, 1375 Gyeongdong-ro, Andong, Gyeongbuk, Republic of Korea, 36729

Contents

**FIGURE S1**	CD spectra of G-quadruplexes for TBA, HTG, PS2.M, and T2 in the presence of Pb^{2+}	1
**FIGURE S2**	Values of the conformational index *r'* for various G-quadruplex with 5, 10, 20 mM K^+ under diluted conditions	2
**FIGURE S3**	CD spectra of TBA G-quadruplexes under K^+ conditions with Pb^{2+} addition in molecular crowding conditions	3
**FIGURE S4**	CD spectra of HTG G-quadruplexes under K^+ conditions with Pb^{2+} addition in molecular crowding conditions	4
**FIGURE S5**	CD spectra of PS2.M G-quadruplexes under K^+ conditions with Pb^{2+} addition in molecular crowding conditions	5
**FIGURE S6**	CD spectra of T2 G-quadruplexes under K^+ conditions with Pb^{2+} addition in molecular crowding conditions	6
**FIGURE S7**	Pb^{2+}-induced displacement of Thioflavin T bound to various G-quadruplexes under molecular crowding conditions	7
**FIGURE S8**	Fluorescence spectra of ThT bound to TBA G-quadruplex in the absence and presence of Pb^{2+}	8
**FIGURE S9**	Fluorescence spectra of ThT bound to HTG G-quadruplex in the absence and presence of Pb^{2+}	9
**FIGURE S10**	Fluorescence spectra of ThT bound to PS2.M G-quadruplex in the absence and presence of Pb^{2+}	10
**FIGURE S11**	Fluorescence spectra of ThT bound to T2 G-quadruplex in the absence and presence of Pb^{2+}	11
**FIGURE S12**	Thermal denaturation curves of various G-quadruplexes in 5 mM K^+ under diluted and molecular crowding conditions	12
**FIGURE S13**	Thermal denaturation curves of various G-quadruplexes in 10 μM Pb^{2+} under diluted and molecular crowding conditions.	13
**FIGURE S14**	Thermal denaturation curves of various G-quadruplexes in 5 mM K^+ with 10 μM Pb^{2+} under diluted and molecular crowding conditions.	14
**FIGURE S15**	CD spectra of various G-quadruplexes in 5 mM K^+ with 10 wt% PEG 200 during titration with Pb^{2+}	15
**FIGURE S16**	CD spectra of various G-quadruplexes in 5 mM K^+ with 30 wt% PEG 200 during titration with Pb^{2+}	16
**FIGURE S17**	Plots of the Langmuir isotherm used to calculate the association constant (K_a) for the G-quadruplexes under molecular crowding conditions	17
**TABLE S1**	Melting temperature data for various G-quadruplexes containing K^+, Pb^{2+}, or with K^+ replaced by Pb^{2+} under diluted and molecular crowding conditions	18

**TABLE S2**	Thermodynamic parameters derived from melting analysis of various G-quadruplexes containing K^+, Pb^{2+}, or K^+ to Pb^{2+} exchange under diluted and molecular crowding conditions	19

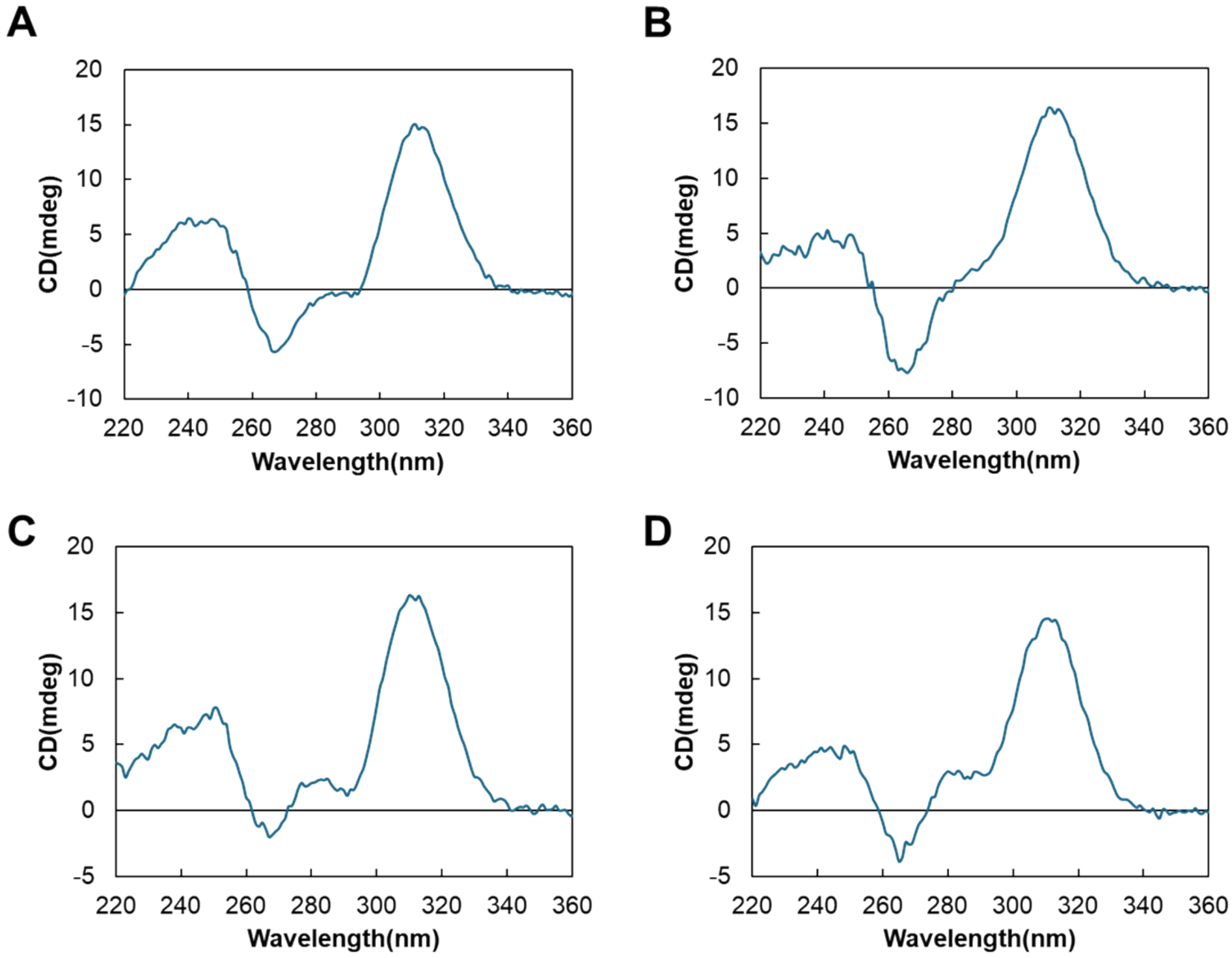

Figure S1. CD spectra of TBA (A), HTG (B), PS2.M (C), and T2 (D) G-quadruplexes in the presence of 3 μM Pb^{2+}. All samples contain 3 μM DNA in 20 mM Tris-HCl buffer (pH 7.0)

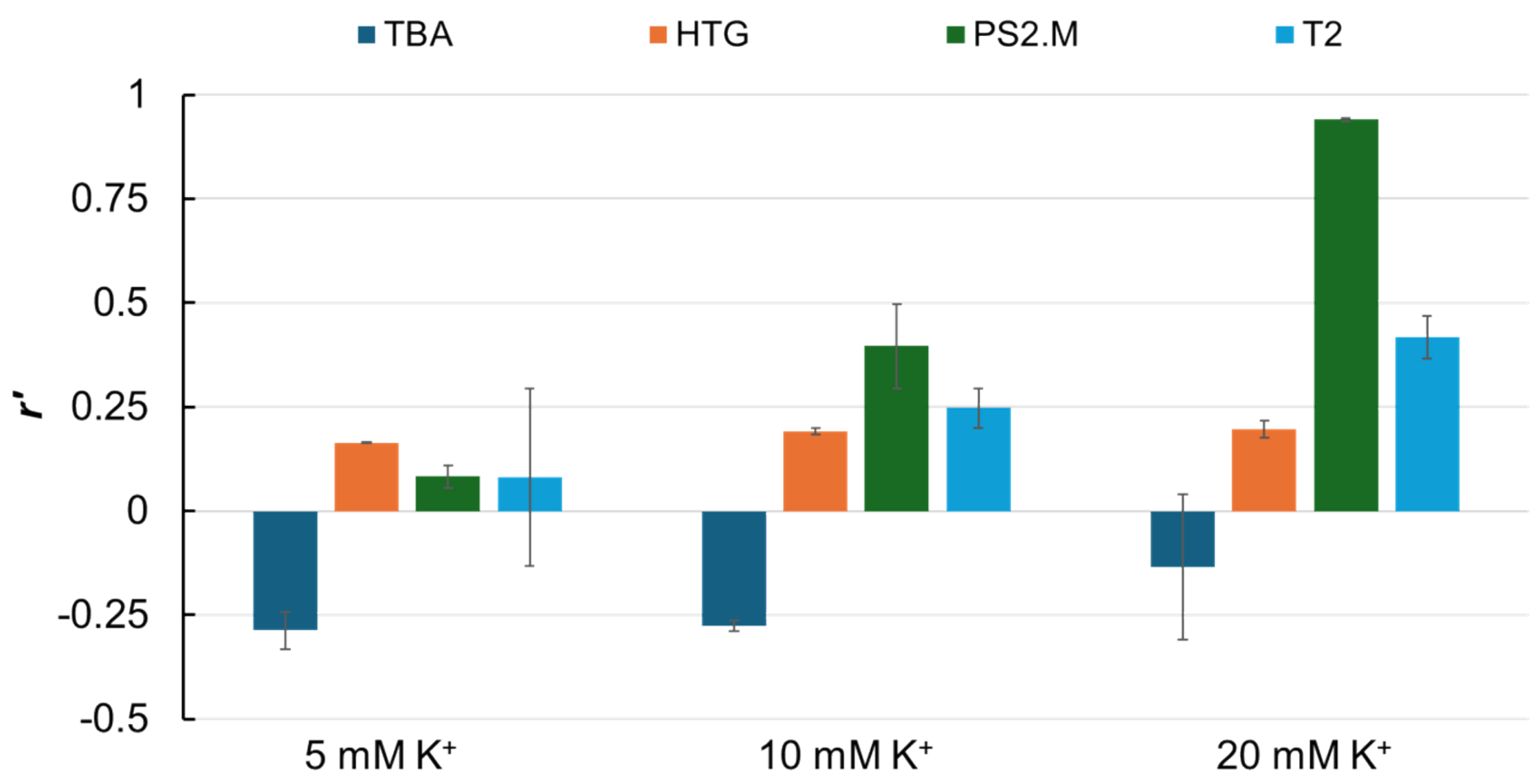

Figure S2. Values of the conformational index *r*' for various G-quadruplex, with TBA, HTG, PS2.M and T2, with 5, 10, and 20 mM K^+. 3 μM DNA was used with appropriate concentration of K^+. All samples contain appropriate concentration of 20 mM Tris-HCl buffer (pH 7.0). The y axis represents the *r'* value ($r' = \frac{CD_{265}}{|CD_{265}| + CD_{290}}$), where CD_{265} and CD_{290} are the CD ellipticities at 265 and 290 nm, respectively.

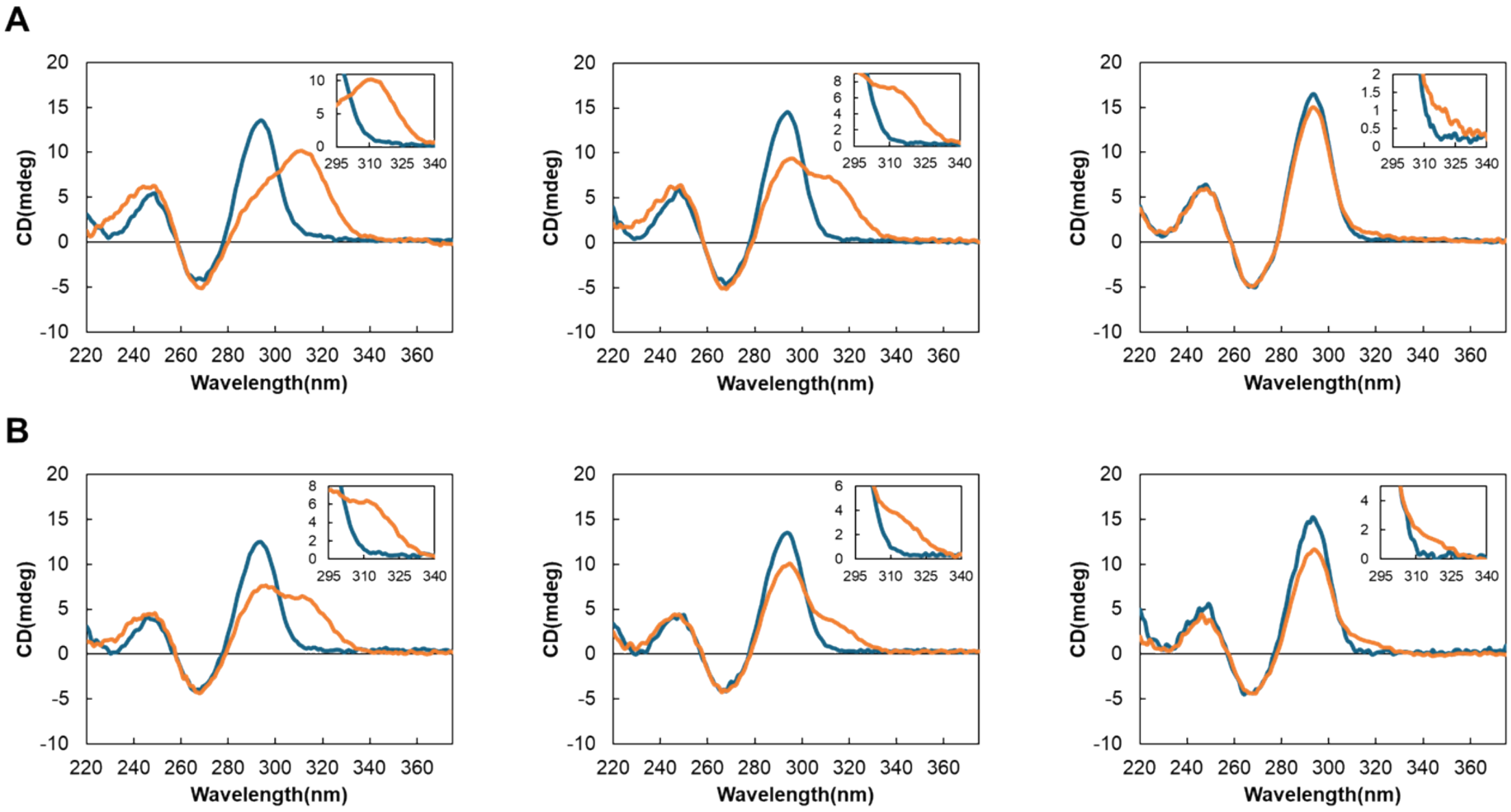

Figure S3. CD spectra of TBA G-quadruplexes containing different concentrations of K^+ (blue line) and affected by 10 μM Pb^{2+} (orange line) under 10 wt% (top) and 30 wt% (bottom) PEG 200. 3 μM of DNA was used, and the samples contain 5 mM K^+ (left panel), 10 mM K^+ (middle panel), and 20 mM K^+ (right panel). Insets show magnified views of the CD spectral region around 314 nm to highlight Pb^{2+}-associated CD signal changes.

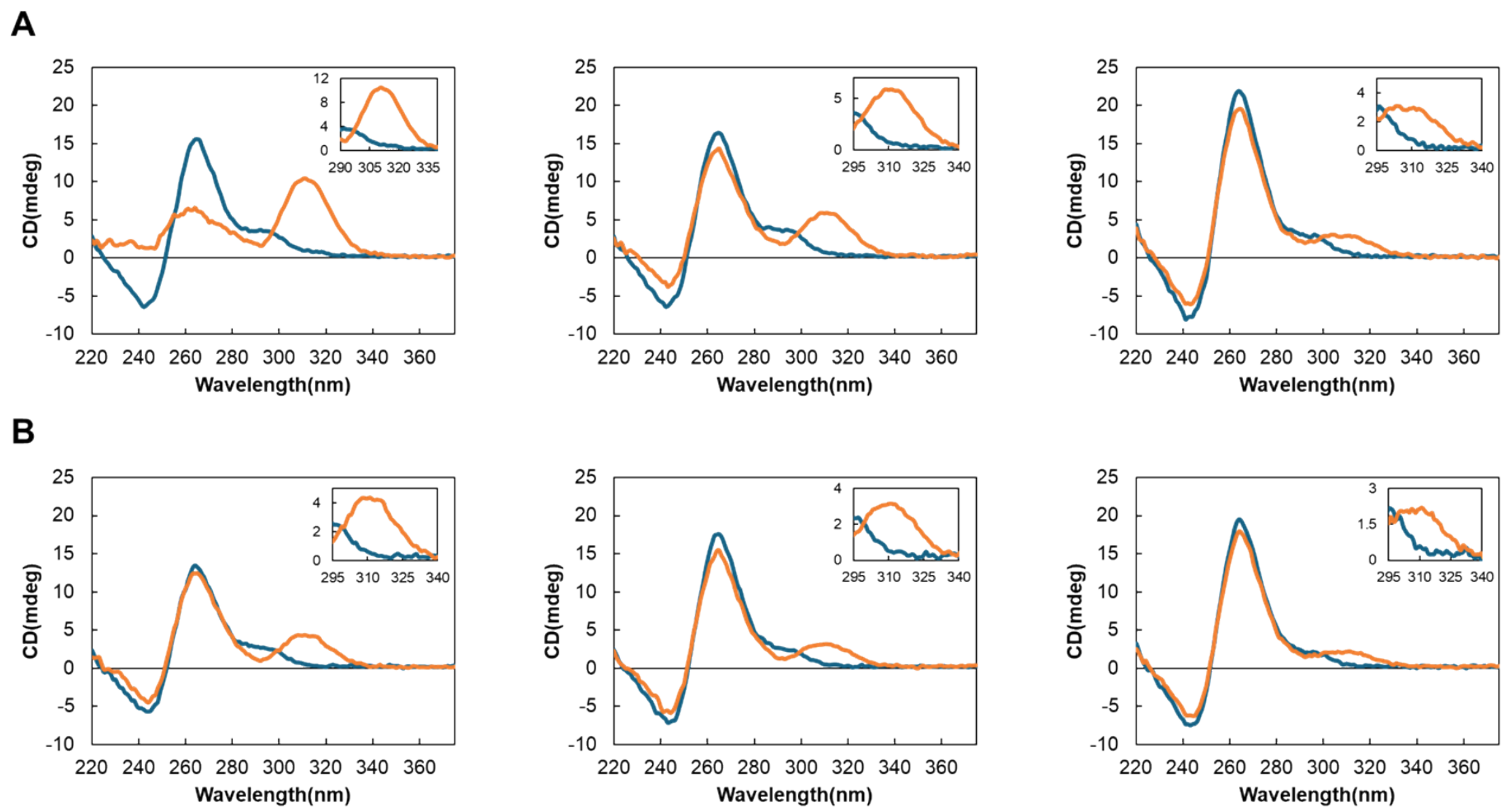

Figure S4. CD spectra of PS2.M G-quadruplexes containing different concentrations of K^+ (blue line) and affected by 10 μM Pb^{2+} (orange line) under 10 wt% (top) and 30 wt% (bottom) PEG 200. 3 μM of DNA was used, and the samples contain 5 mM K^+ (left panel), 10 mM K^+ (middle panel), and 20 mM K^+ (right panel). Insets show magnified views of the CD spectral region around 314 nm to highlight Pb^{2+}-associated CD signal changes.

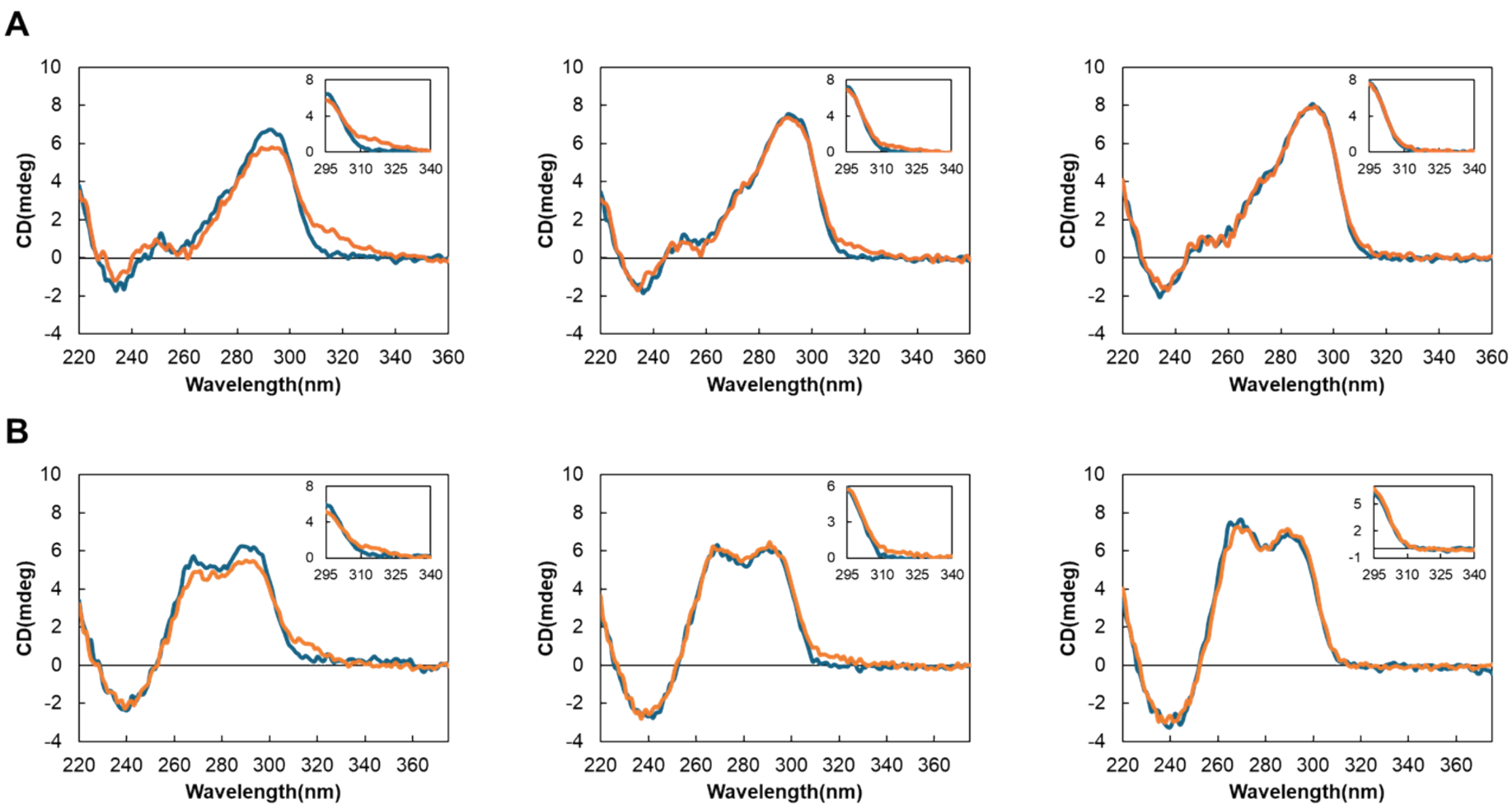

Figure S5. CD spectra of HTG G-quadruplexes containing different concentrations of K^+ (blue line) and affected by 10 μM Pb^{2+} (orange line) under 10 wt% (A) and 30 wt% (B) PEG 200. 3 μM of DNA was used, and the samples contain 5 mM K^+ (left panel), 10 mM K^+ (middle panel), and 20 mM K^+ (right panel). Insets show magnified views of the CD spectral region around 314 nm to highlight Pb^{2+}-associated CD signal changes.

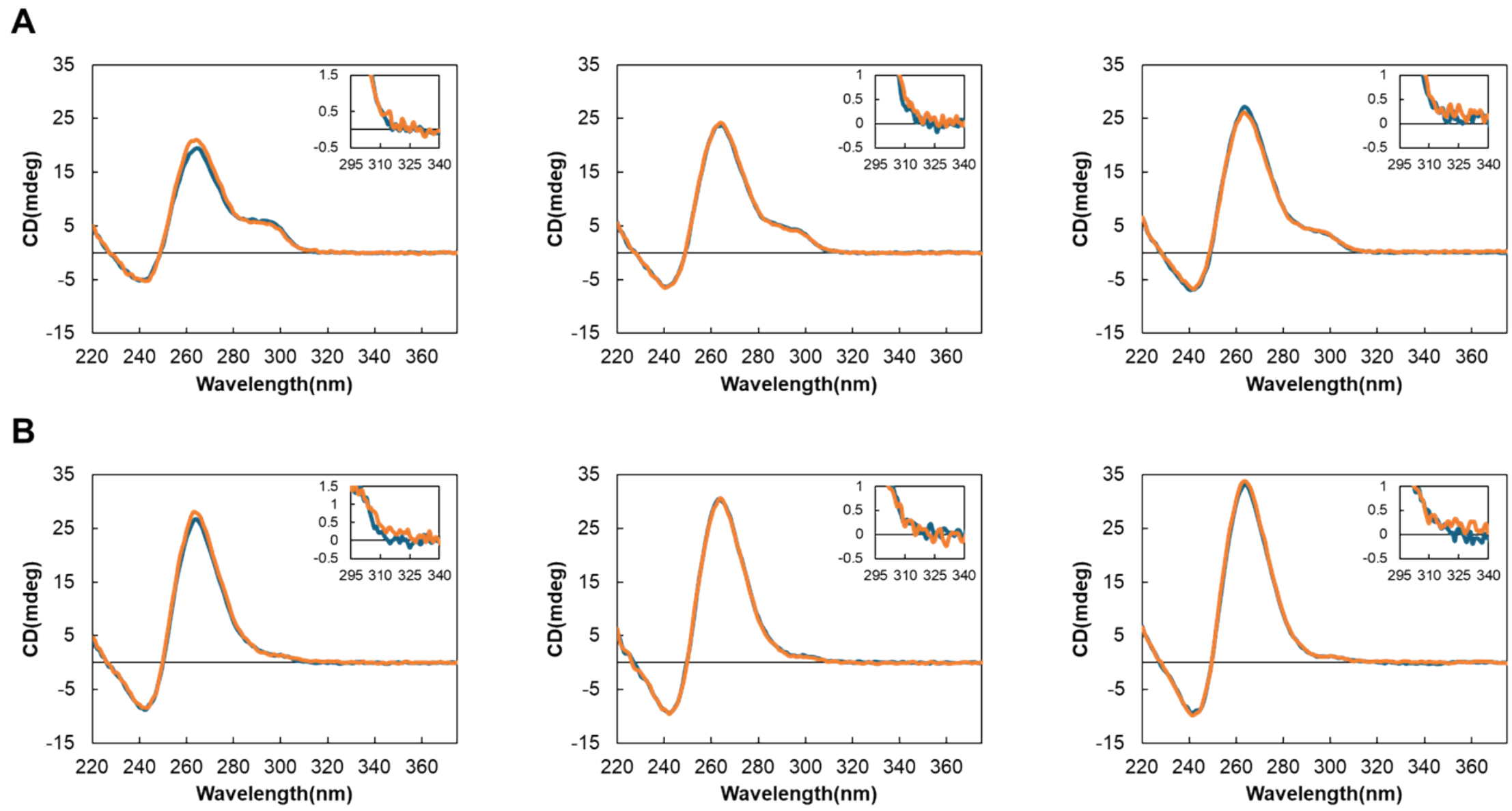

Figure S6. CD spectra of T2 G-quadruplexes containing different concentrations of K^+ (blue line) and affected by 10 μM Pb^{2+} (orange line) under 10 wt% (A) and 30 wt% (B) PEG 200. 3 μM of DNA was used and the samples contain 5 mM K^+ (left panel), 10 mM K^+ (middle panel), and 20 mM K^+ (right panel). Insets show magnified views of the CD spectral region around 314 nm to highlight Pb^{2+}-associated CD signal changes.

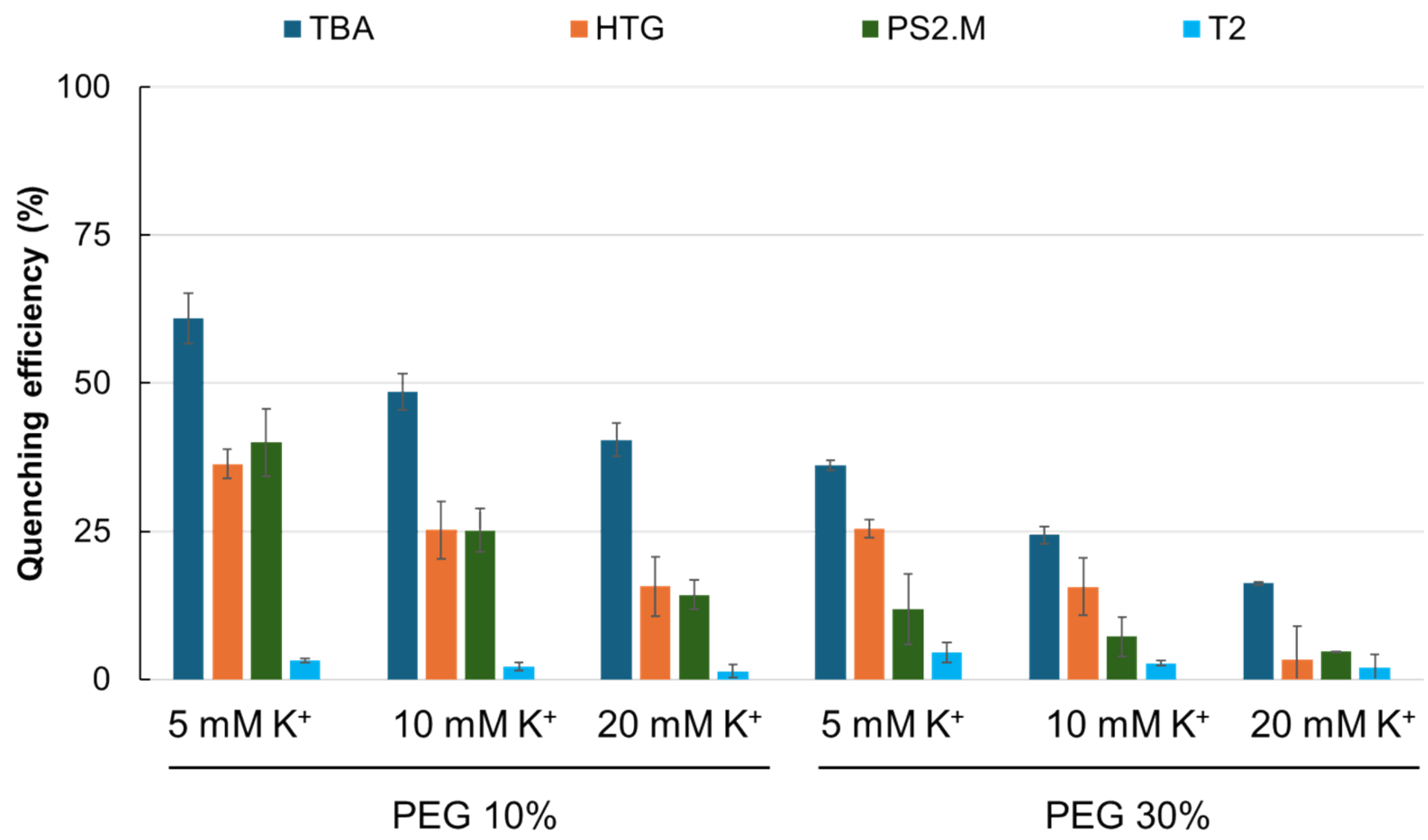

Figure S7. Fluorescence quenching efficiency of various G-quadruplex by Pb^{2+} under molecular crowding condition. 3 μM DNA was used with an appropriate concentration of K^{+} in presence of 6 μM ThT and with addition of 10 μM Pb^{2+}. All samples contain appropriate concentration of 20 mM Tris-HCl buffer (pH 7.0).

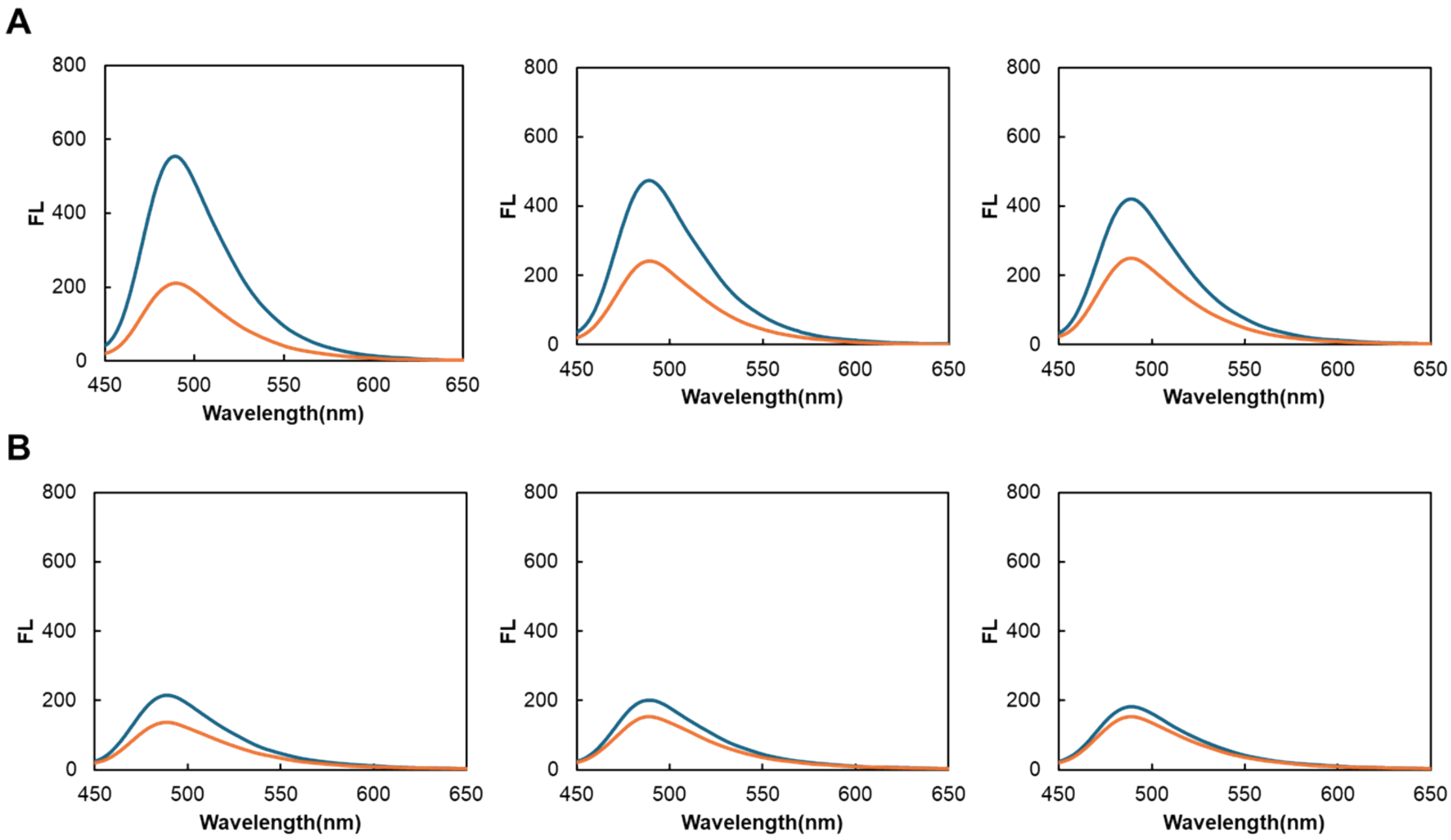

Figure S8. Fluorescence spectra of 6 μM ThT bound to 3 μM TBA G-quadruplex containing 5 (left panel), 10 (middle panel), and 20 mM (right panel) K^+ in the absence (dark blue) and presence (orange) of 10 μM Pb^{2+}. All samples contain an appropriate concentration of 20 mM Tris-HCl buffer (pH 7.0) in presence of 10 wt% (A) and 30 wt% (B) PEG 200.

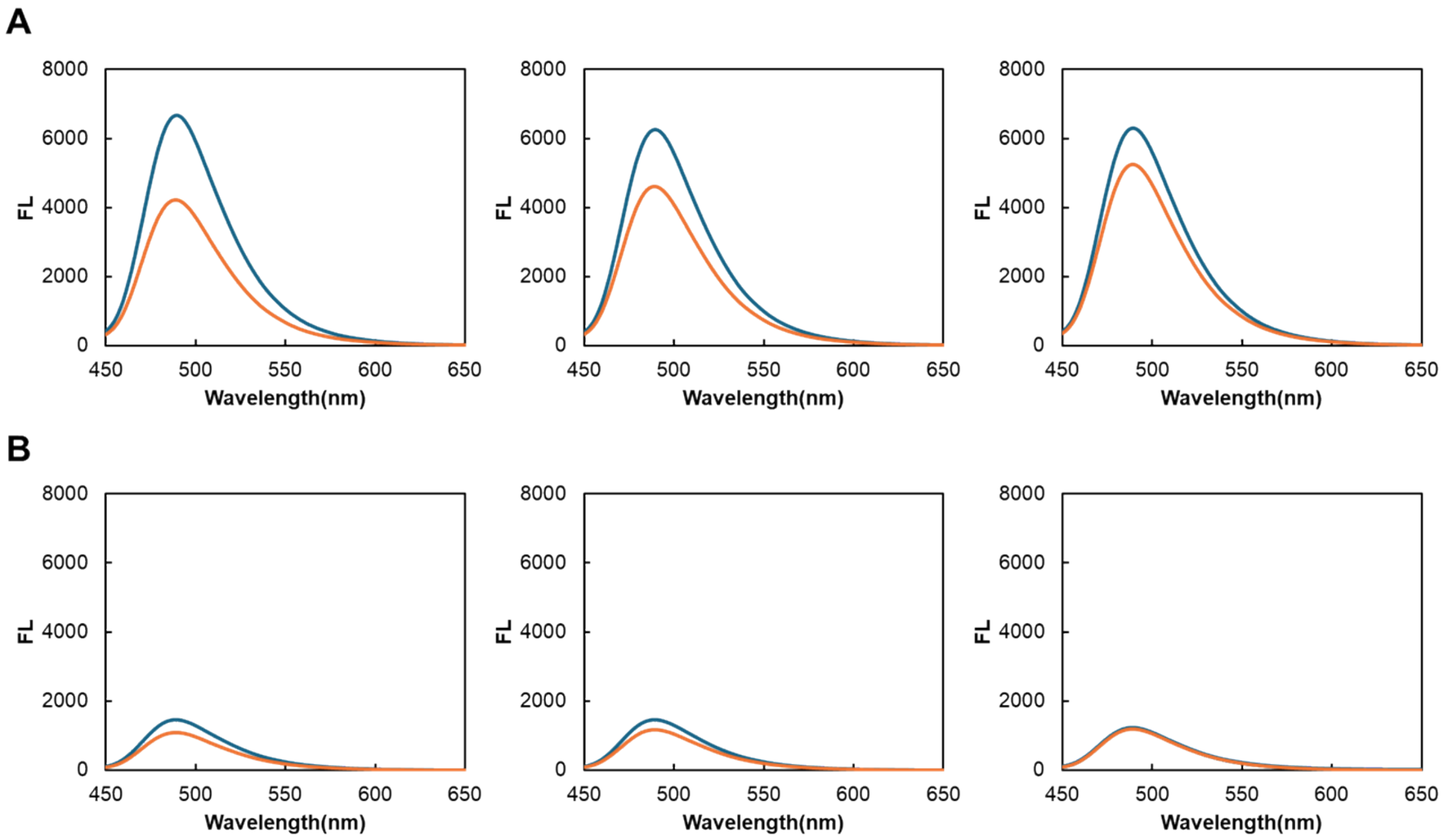

Figure S9. Fluorescence spectra of 6 μM ThT bound to 3 μM HTG G-quadruplex containing 5 (left panel), 10 (middle panel), and 20 mM (right panel) K^+ in the absence (dark blue) and presence (orange) of 10 μM Pb^{2+}. All samples contain an appropriate concentration of 20 mM Tris-HCl buffer (pH 7.0) in presence of 10 wt% (A) and 30 wt% (B) PEG 200.

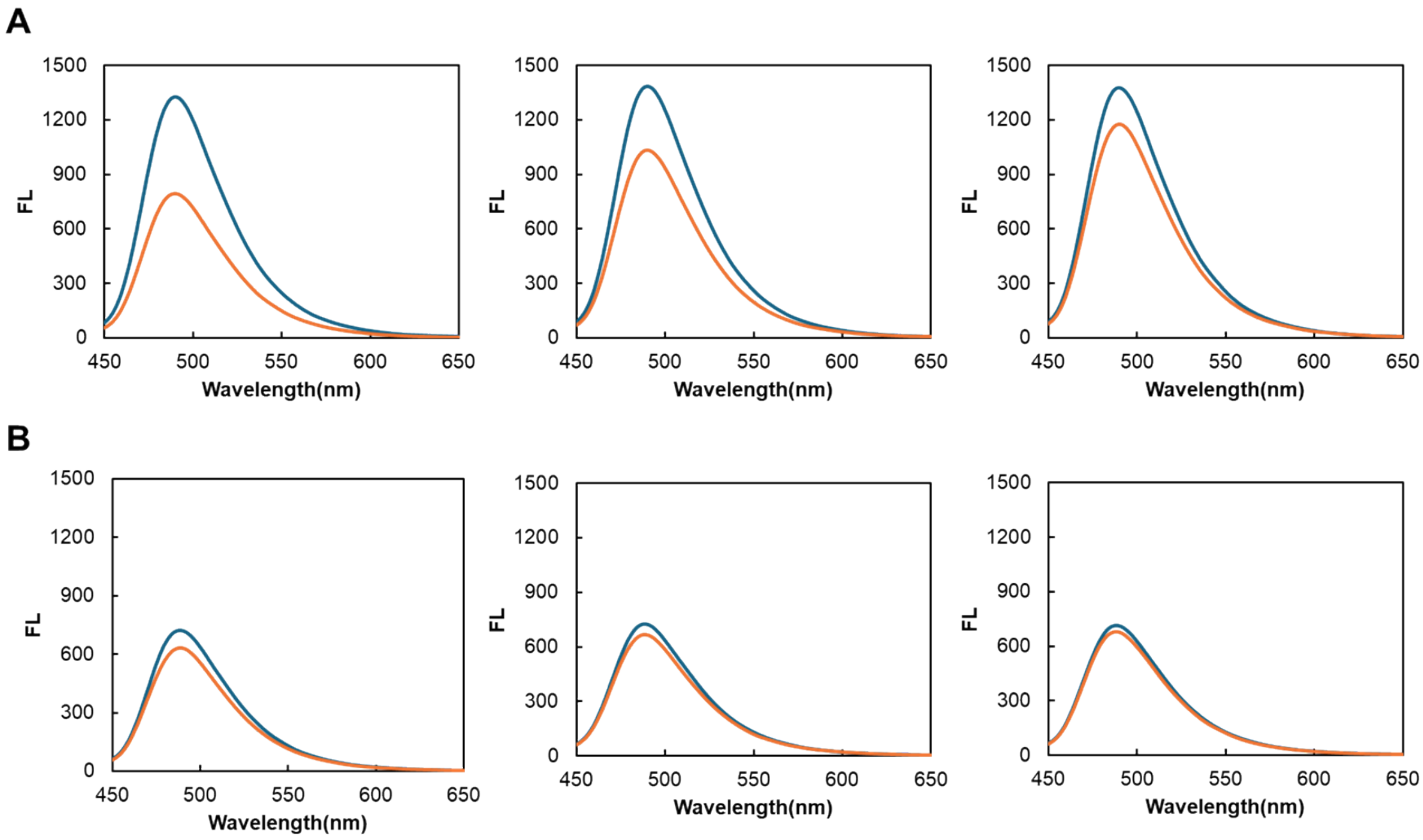

Figure S10. Fluorescence spectra of 6 μM ThT bound to 3 μM PS2.M G-quadruplex containing 5 (left panel), 10 (middle panel), and 20 mM (right panel) K^+ in the absence (dark blue) and presence (orange) of 10 μM Pb^{2+}. All samples contain an appropriate concentration of 20 mM Tris-HCl buffer (pH 7.0) in presence of 10 wt% (A) and 30 wt% (B) PEG 200.

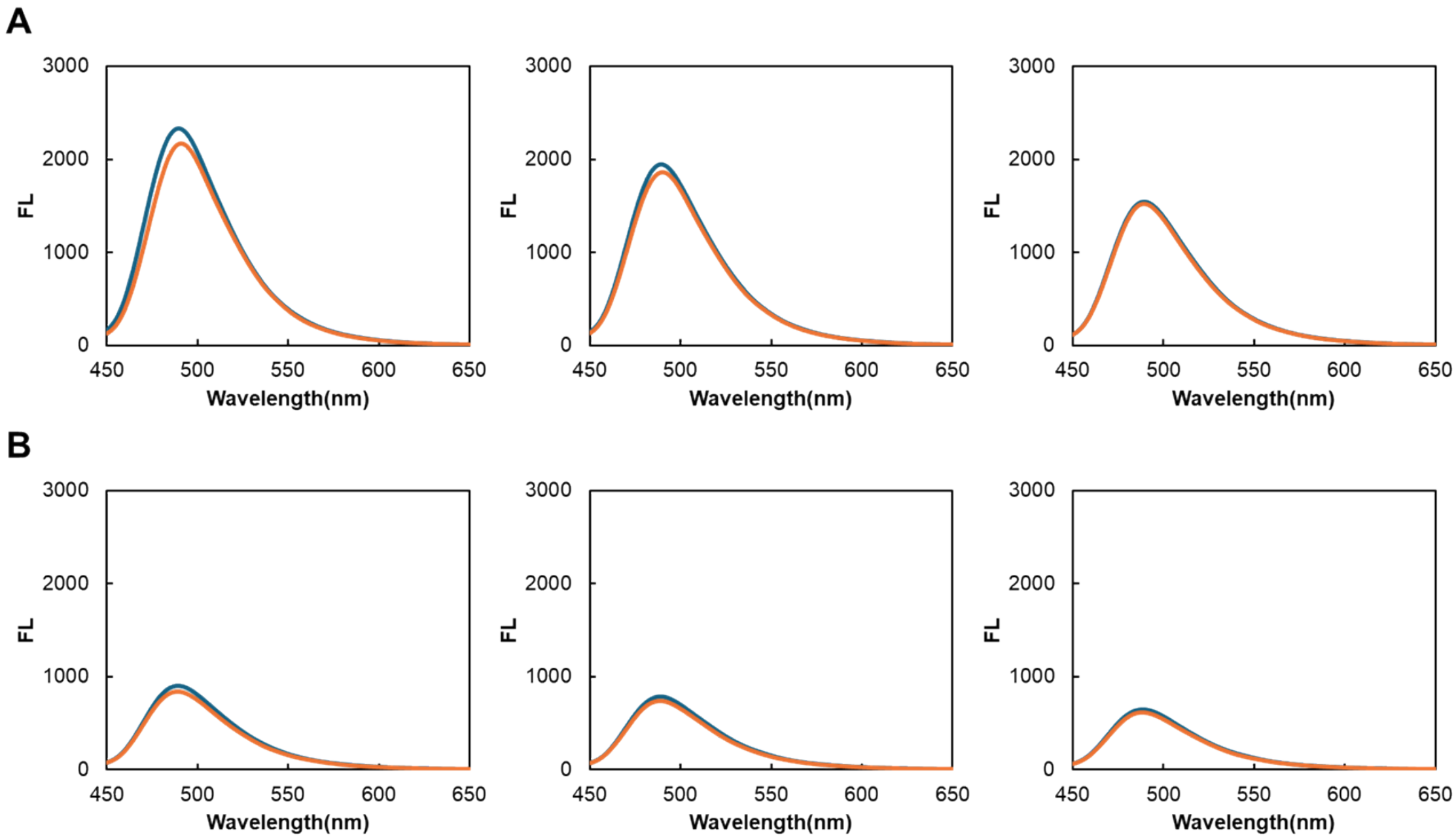

Figure S11. Fluorescence spectra of 6 μM ThT bound to 3 μM T2 G-quadruplex containing 5 (left panel), 10 (middle panel), and 20 mM (right panel) K^+ in the absence (dark blue) and presence (orange) of 10 μM Pb^{2+}. All samples contain an appropriate concentration of 20 mM Tris-HCl buffer (pH 7.0) in presence of 10 wt% (A) and 30 wt% (B) PEG 200.

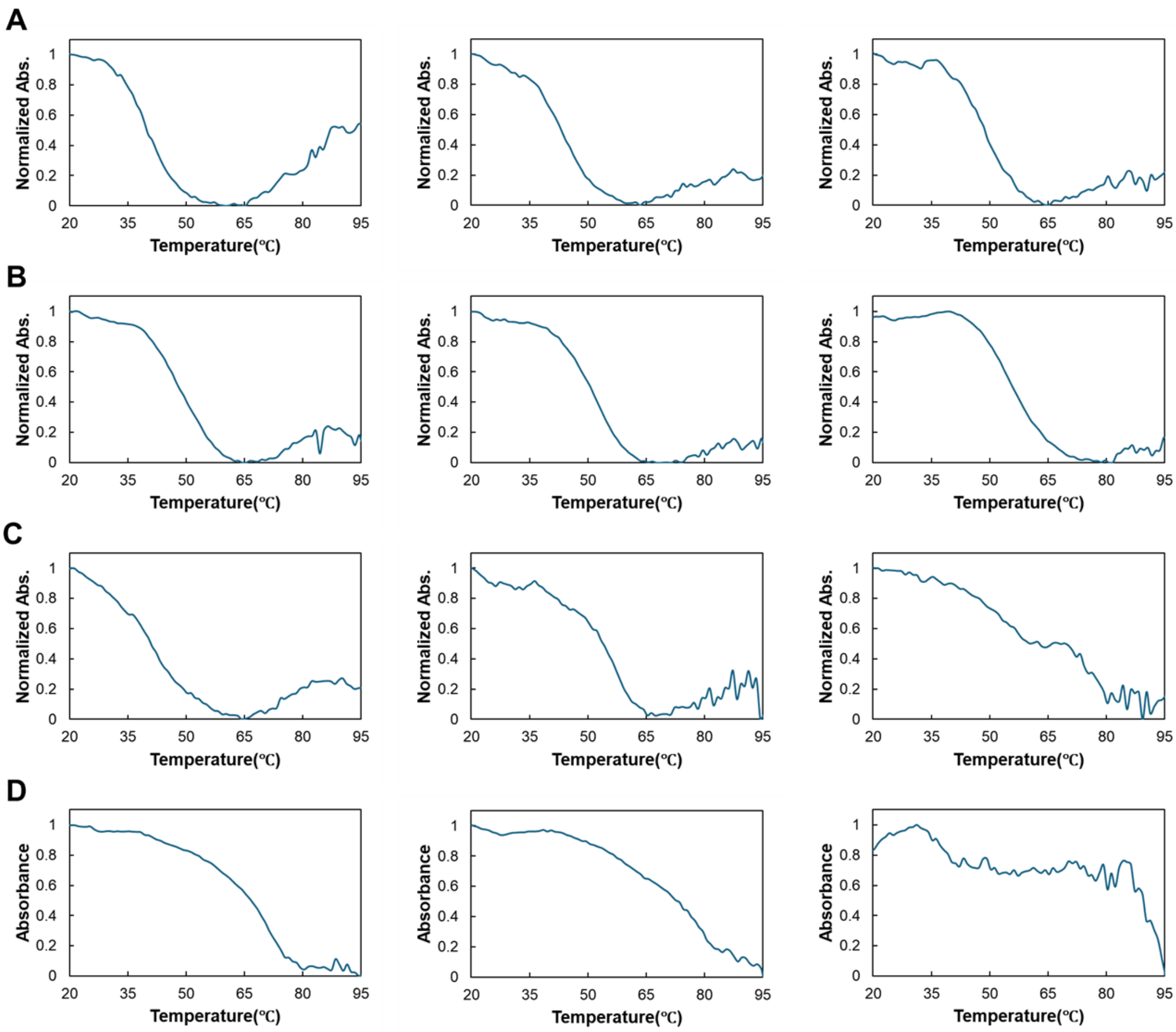

Figure S12. Thermal denaturation curves of various G-quadruplex, with TBA (A), HTG (B), PS2.M (C), and T2 (D) in 5 mM K^+ under diluted condition (left), 10 wt% (middle) and 30 wt% (right) PEG 200 as molecular crowding conditions. 3 μM DNA was used in 20 mM Tris-HCl buffer (pH 7.0).

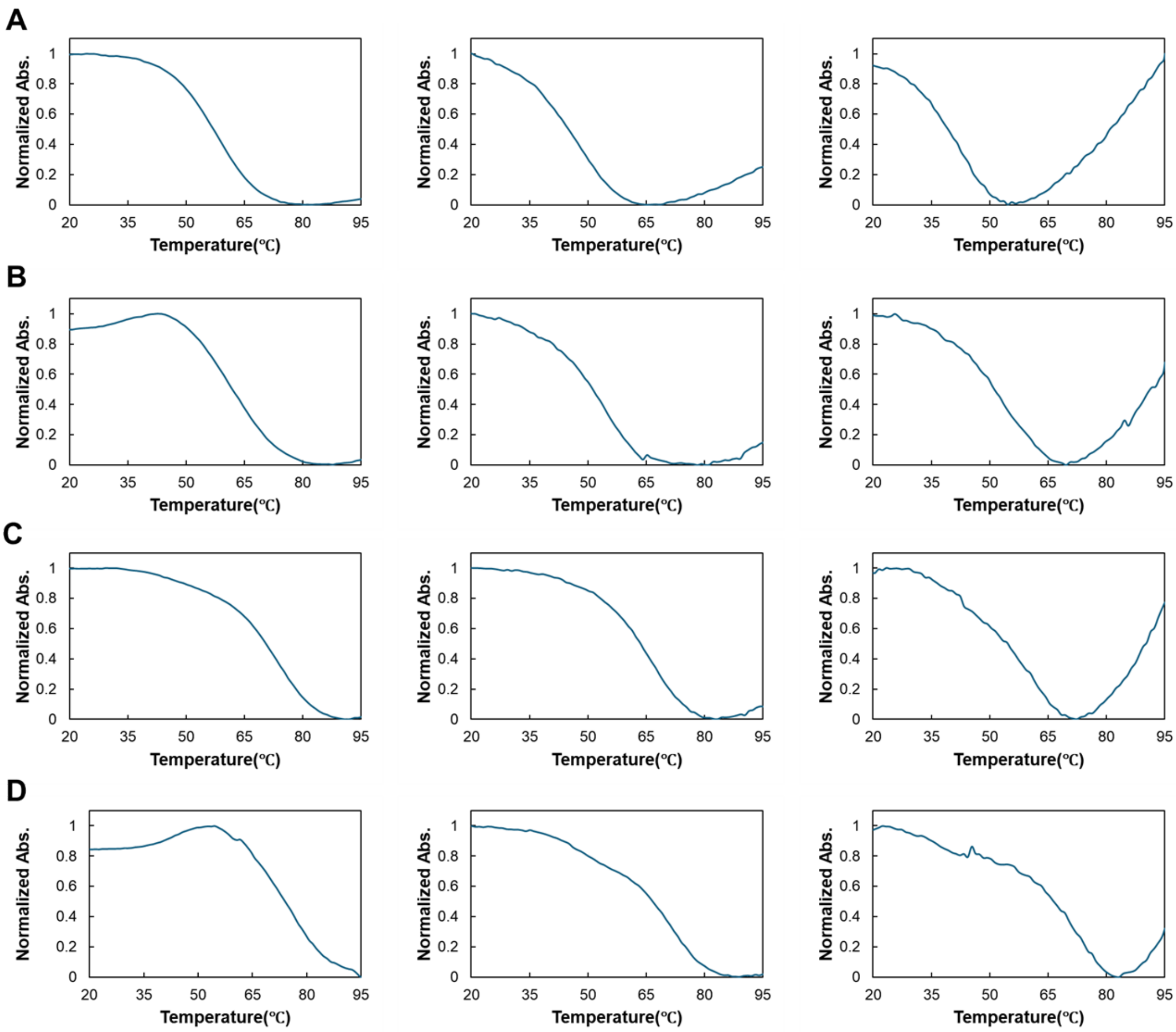

Figure S13. Thermal denaturation curves of various G-quadruplexes, with TBA (A), HTG (B), PS2.M (C), and T2 (D) in 10 μM Pb^{2+} under diluted condition (left), 10 wt% (middle), and 30 wt% (right) PEG 200 as molecular crowding conditions. 3 μM DNA was used in 20 mM Tris-HCl buffer (pH 7.0).

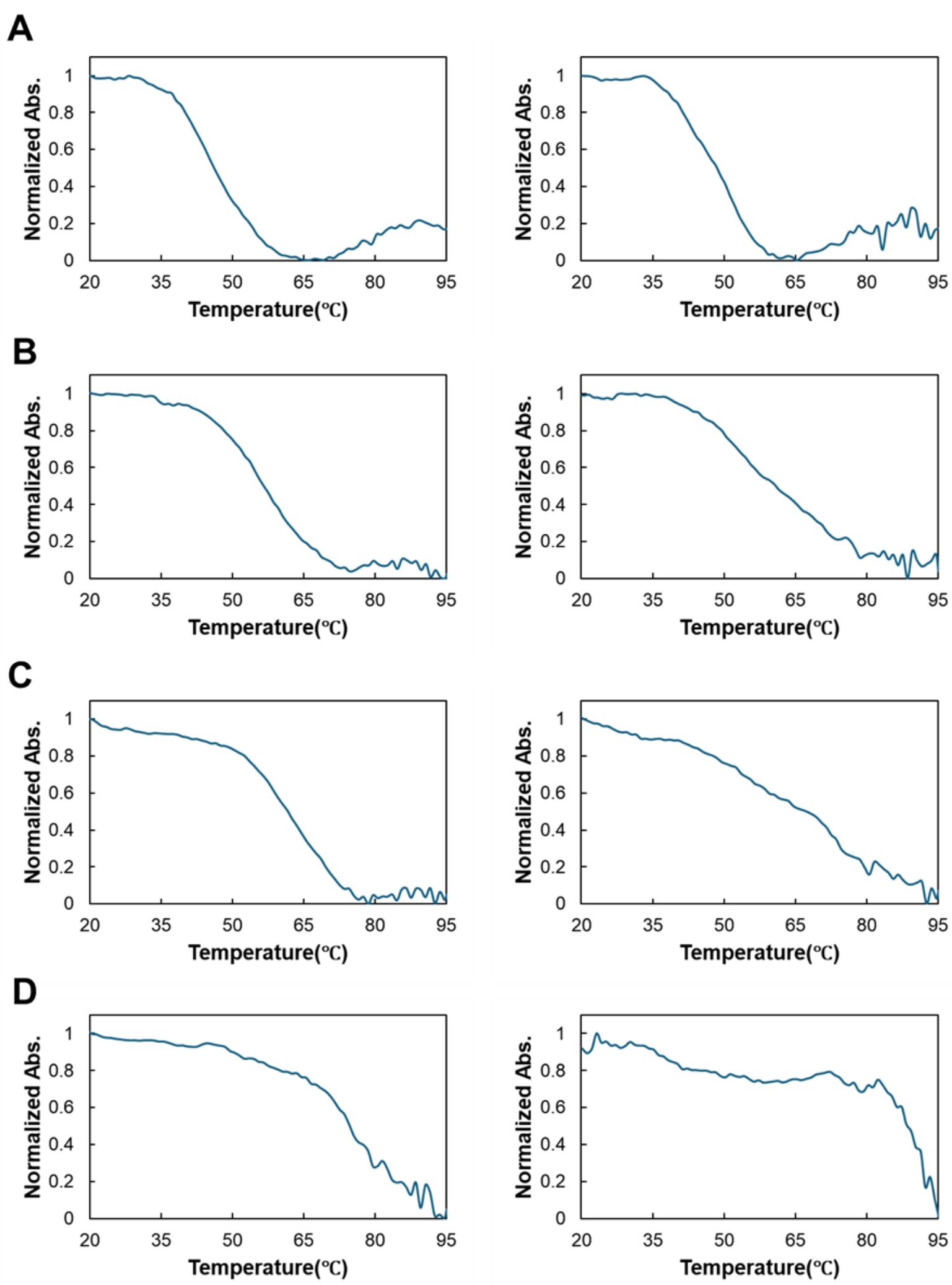

Figure S14. Thermal denaturation curves of various G-quadruplexes, with TBA (A), HTG (B), PS2.M (C), and T2 (D), in 5 mM K^+ after addition of 10 μM Pb^{2+} under diluted condition (left), 10 wt% (middle), and 30 wt% (right) PEG 200 as molecular crowding conditions. 3 μM DNA was used in 20 mM Tris-HCl buffer (pH 7.0).

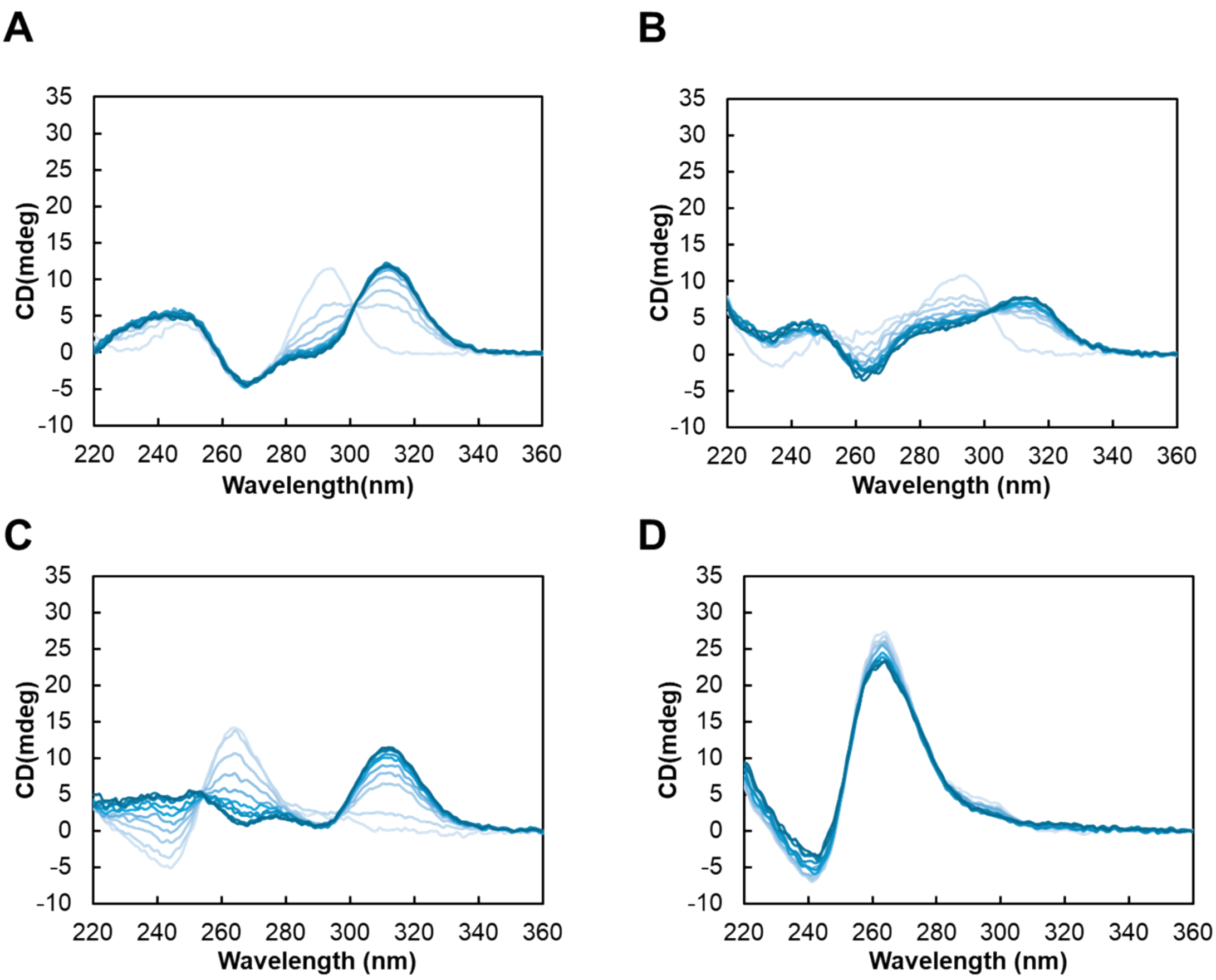

Figure S15. CD spectra of G-quadruplexes TBA (A), HTG (B), PS2.M (C), and T2 (D), each containing 5 mM K^+ under 10 wt% PEG 200, were obtained during titration with 10 μM Pb^{2+} (10, 20, 30, 40, 50, 60, 70, 80, 90, and 100 μM). The spectra are shown in progressively darker blue as the Pb^{2+} concentration increases. All samples contained 3 μM DNA with appropriate concentrations of cations and PEG 200 in the presence of 20 mM Tris-HCl buffer (pH 7.0).

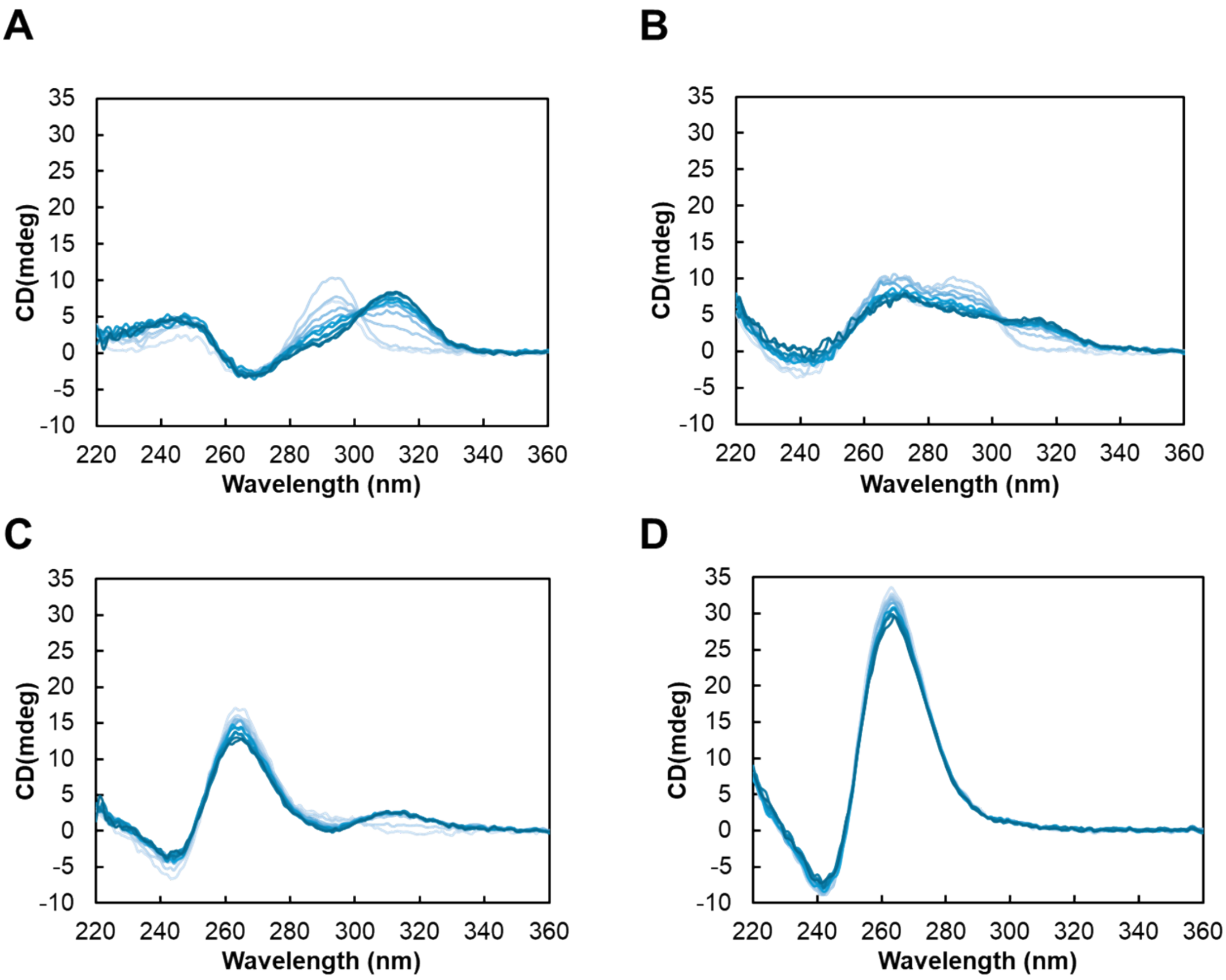

Figure S16. CD spectra of G-quadruplexes TBA (A), HTG (B), PS2.M (C), and T2 (D), each containing 5 mM K^+ under 30 wt% PEG 200, were obtained during titration with 10 µM Pb^{2+} (10, 20, 30, 40, 50, 60, 70, 80, 90, and 100 µM). The spectra are shown in progressively darker blue as the Pb^{2+} concentration increases. All samples contained 3 µM DNA with appropriate concentrations of cations and PEG 200 in the presence of 20 mM Tris-HCl buffer (pH 7.0).

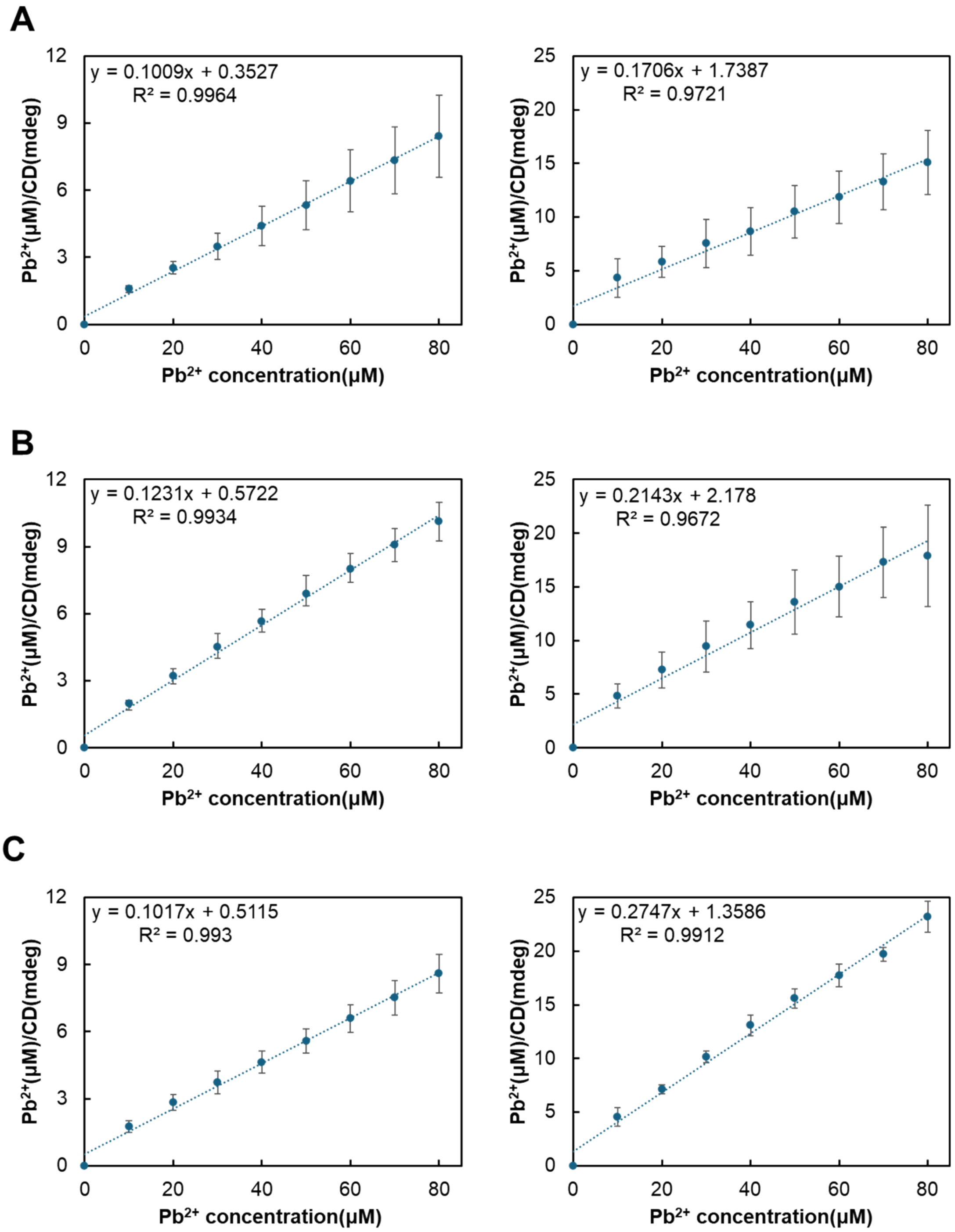

Figure S17. Plots of the Langmuir isotherm used to calculate the association constant (K_a) for the G-quadruplexes TBA (A), HTG (B), and PS2.M (C) titrated with Pb^{2+} under 10 wt% (left panel) and 30 wt% PEG 200 (right panel). The plots were obtained by plotting *c* versus *c*/*q*. The plotted data represents the average of three independent

measurements.

Table S1. Melting temperature (T_m) data for various G-quadruplexes containing K^+, Pb^{2+}, or with K^+ replaced by Pb^{2+} under 10 wt% and 30 wt% PEG 200.

	**Only K^+**			**Only Pb^{2+}**			**$K^+ \rightarrow Pb^{2+}$**	
	Diluted	10 wt% PEG 200	30 wt% PEG200	Diluted	10 wt% PEG 200	30 wt% PEG200	10 wt% PEG 200	30 wt% PEG200
**TBA**	42.6±2.5	43.1±1.0	51.0±2.1	58.5±1.3	46.5±0.3	40.6±0.3	47.2±0.6	51.4±3.2
**HTG**	48.4±0.5	51.3±0.3	56.7±1.6	61.7±0.3	56.6±5.1	48.5±1.1	54.3±2.1	58.0±0.7
**PS2.M**	41.4±0.5	54.8±0.9	70.7±0.2	72.3±0.3	64.2±1.2	61.7±0.1	63.2±0.1	67.1±0.1
**T2**	66.3±0.1	70.4±2.1	N.A	73.1±0.1	77.9±1.9	75.7±0.4	77.1±1.2	N.A

Table S2. Thermodynamic parameters derived from melting analysis for the unfolding transition of various G-quadruplex structures to ssDNA in the presence of K^+, Pb^{2+}, or K^+-to-Pb^{2+} exchange under 10 wt% and 30 wt% PEG 200 conditions.

		**Only K^+**			**Only Pb^{2+}**			**$K^+ \rightarrow Pb^{2+}$**		
	**PEG**	**$\Delta H°$ (kJ mol^{-1})**	**$\Delta S°$ (J mol^{-1}K^{-1})**	**$\Delta G°_{(37)}$ (kJ mol^{-1})**	**$\Delta H°$ (kJ mol^{-1})**	**$\Delta S°$ (J mol^{-1}K^{-1})**	**$\Delta G°_{(37)}$ (kJ mol^{-1})**	**$\Delta H°$ (kJ mol^{-1})**	**$\Delta S°$ (J mol^{-1}K^{-1})**	**$\Delta G°_{(37)}$ (kJ mol^{-1})**
**TBA**	**Diluted**	196.4±0.7	618.7±6.7	4.5±2.8	151.5±5.3	457.3±17.7	9.7±0.2	N.M.	N.M.	N.M.
	**10 wt%**	188.1±4.9	596.0±15.0	3.3±0.3	153.6±1.0	483.5±3.4	3.7±0.1	186.5±1.8	581.6±7.7	6.1±0.6
	**30 wt%**	238.0±1.8	732.1±2.2	10.9±2.5	159.7±0.0	509.0±0.4	1.8±0.2	162.6±2.0	500.3±0.8	7.5±1.8
**HTG**	**Diluted**	171.8±2.0	535.9±5.0	5.5±0.4	162.9±6.1	486.7±17.7	11.9±0.6	N.M.	N.M.	N.M.
	**10 wt%**	131.0±2.8	408.9±5.1	4.2±1.2	122.2±3.5	372.0±4.8	6.8±2.0	166.2±4.3	507.8±16.4	8.7±0.8
	**30 wt%**	89.9±0.2	276.9±3.0	4.0±1.1	155.1±3.8	481.0±13.8	6.0±0.5	106.2±1.7	322.0±3.7	6.4±0.6
**PS2.M**	**Diluted**	185.3±3.0	588.7±7.7	2.7±0.6	172.4±1.0	502.6±3.1	16.5±0.0	N.M.	N.M.	N.M.
	**10 wt%**	218.0±5.4	664.0±17.3	12.1±0.0	147.9±2.7	440.3±7.1	11.3±0.4	201.7±5.2	600.3±15.7	15.6±0.3
	**30 wt%**	378.5±3.6	1052.3±11.9	52.1±0.1	193.9±4.4	575.9±7.3	15.3±2.1	116.7±1.1	343.9±4.1	10.0±0.1
**T2**	**Diluted**	367.8±13.5	1053.9±12.8	41.0±9.5	171.3±1.6	494.8±3.9	17.8±0.4	N.M.	N.M.	N.M.
	**10 wt%**	138.0±7.7	393.0±15.8	16.1±2.8	182.3±2.8	529.4±2.0	18.1±2.1	149.4±5.1	430.5±13.2	15.9±1.0
	**30 wt%**	N.D.	N.D.	N.D.	232.2±6.2	670.6±12.5	24.2±2.4	N.D.	N.D.	N.D.

$\Delta G°_{(37)}$ represents the standard Gibbs free energy change at 37 °C. N.M., not measured under the corresponding condition; N.D., not determined due to unreliable melting analysis.
